# Supplementary material for: Synthesis, Anti-Inflammatory, and Molecular Docking Studies of New Heterocyclic Derivatives Comprising Pyrazole, Pyridine, and/or Pyran Moieties
Source: Pharmaceuticals (Basel). 2025 Feb 26;18(3):335. doi: 10.3390/ph18030335 (PMC11944836; doi:10.3390/ph18030335)
Supplement: Supplementary file 1 [file pharmaceuticals-18-00335-s001.zip › pharmaceuticals-3462701-supplementary.pdf]

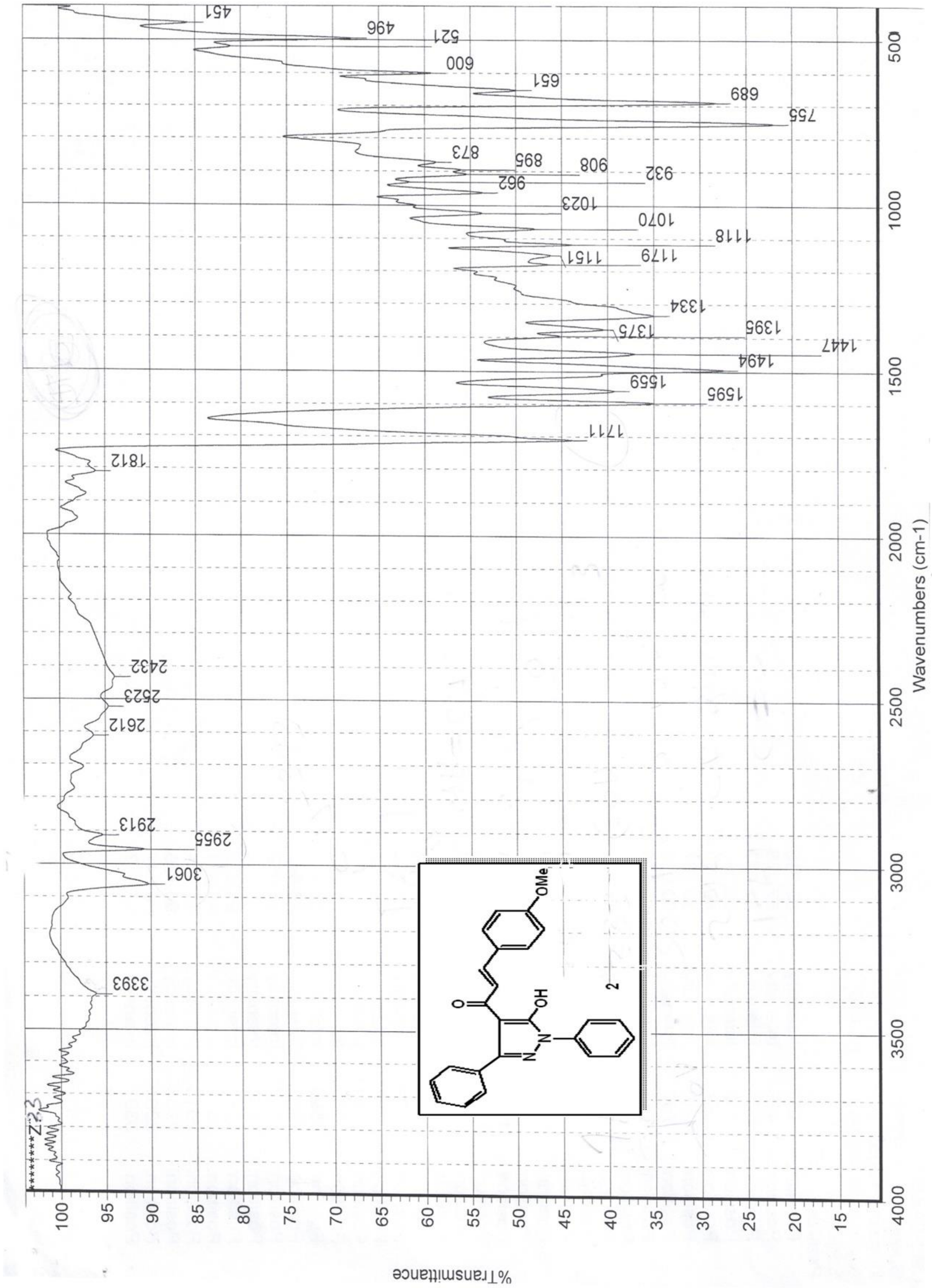

Figure S1: IR Spectrum of Compound (2)

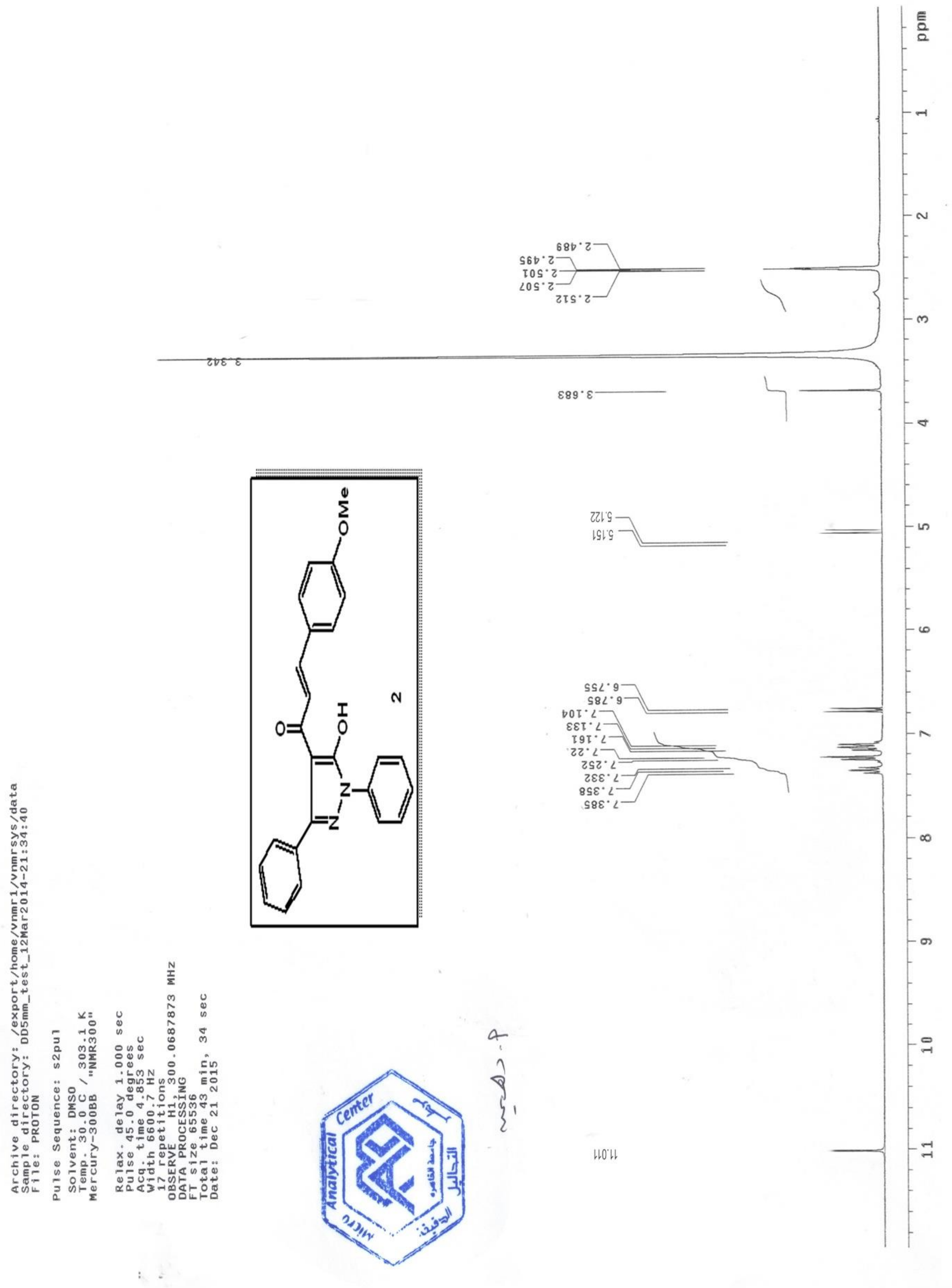

Figure S2: ^{1}H-NMR Spectrum of Compound (2) ….(DMSO)

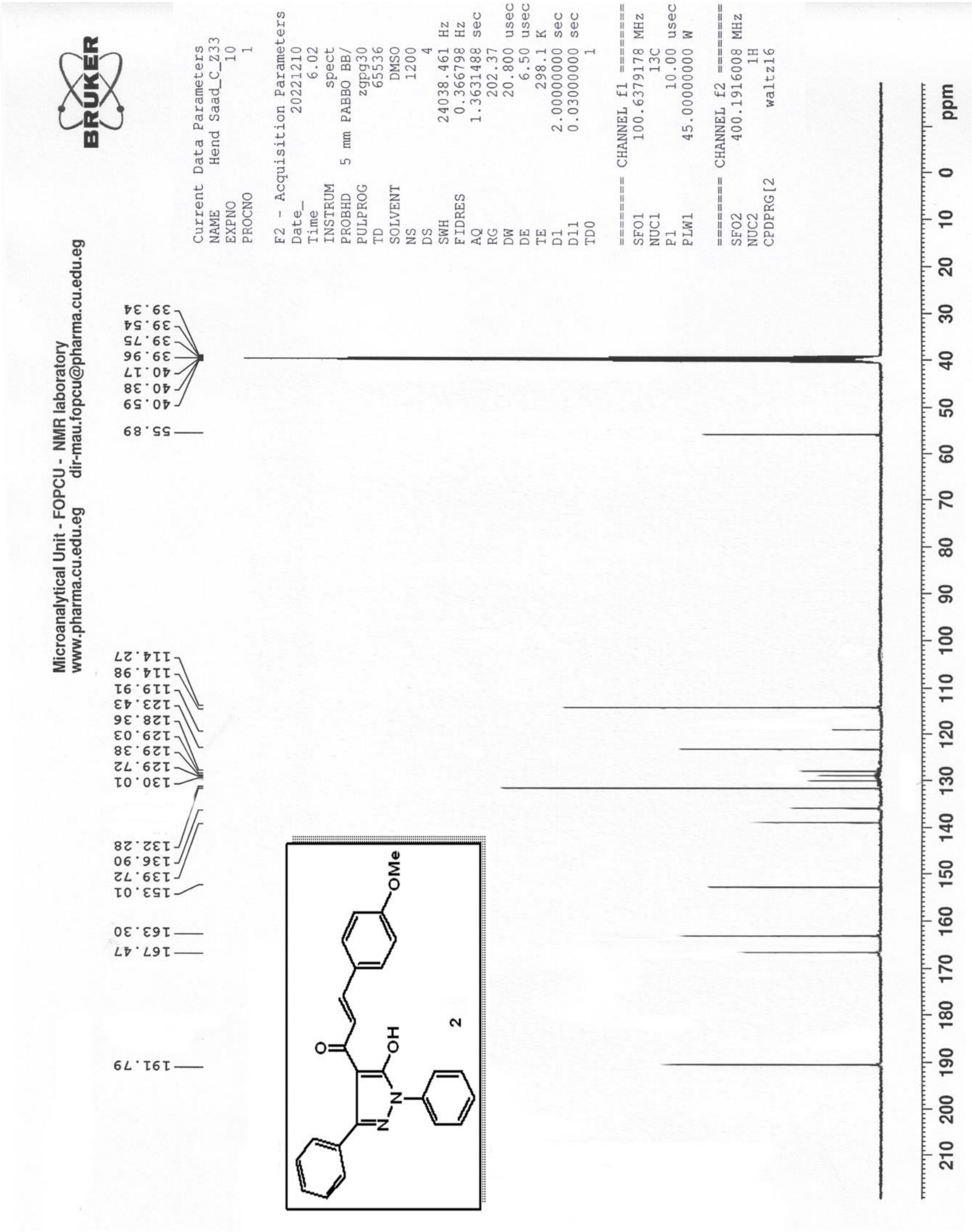

Figure S3: ^{13}C-NMR Spectrum of Compound (2)…… (DMSO)

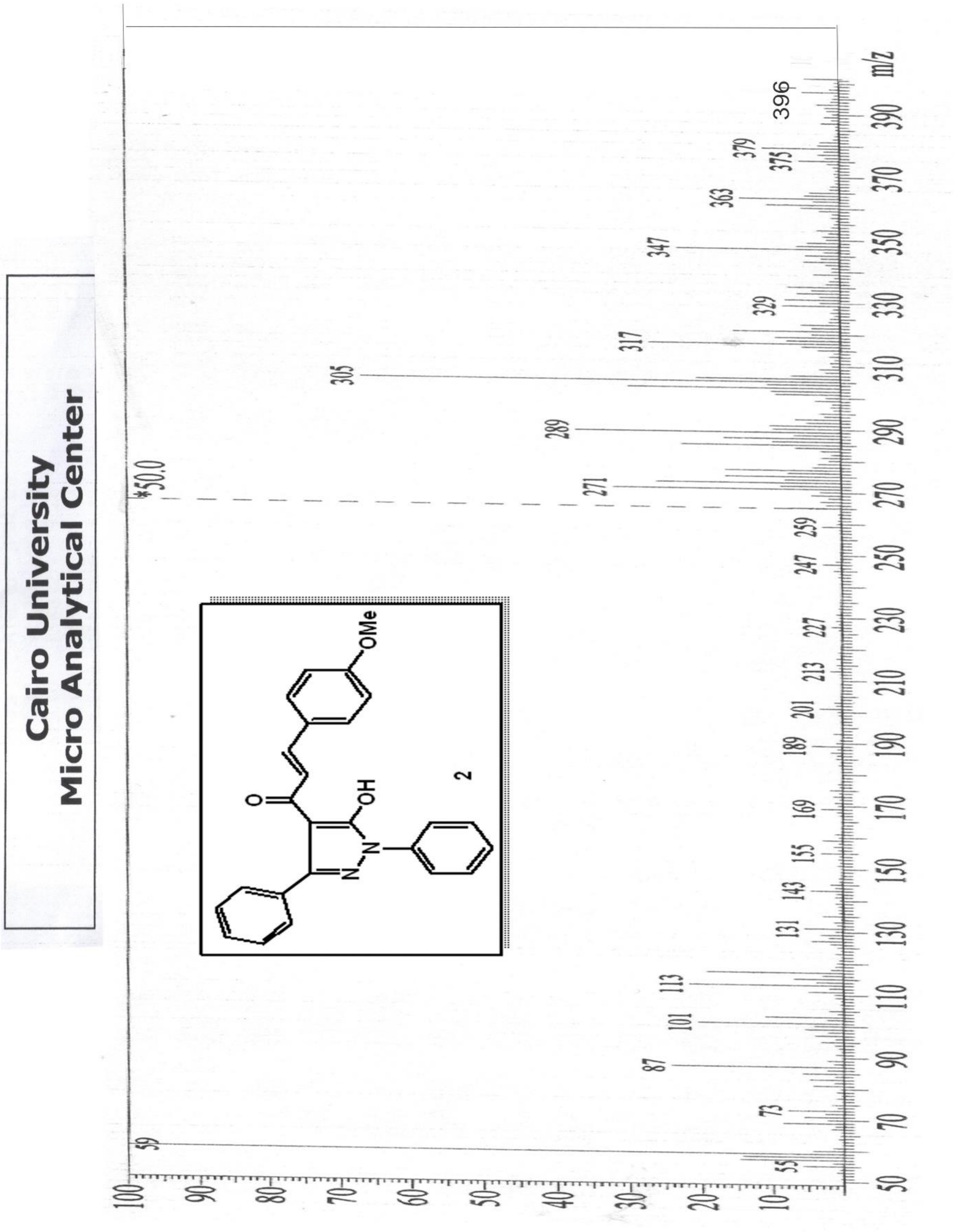

Figure S4: Mass Spectrum of Compound (2) M.wt=396 (M^+)

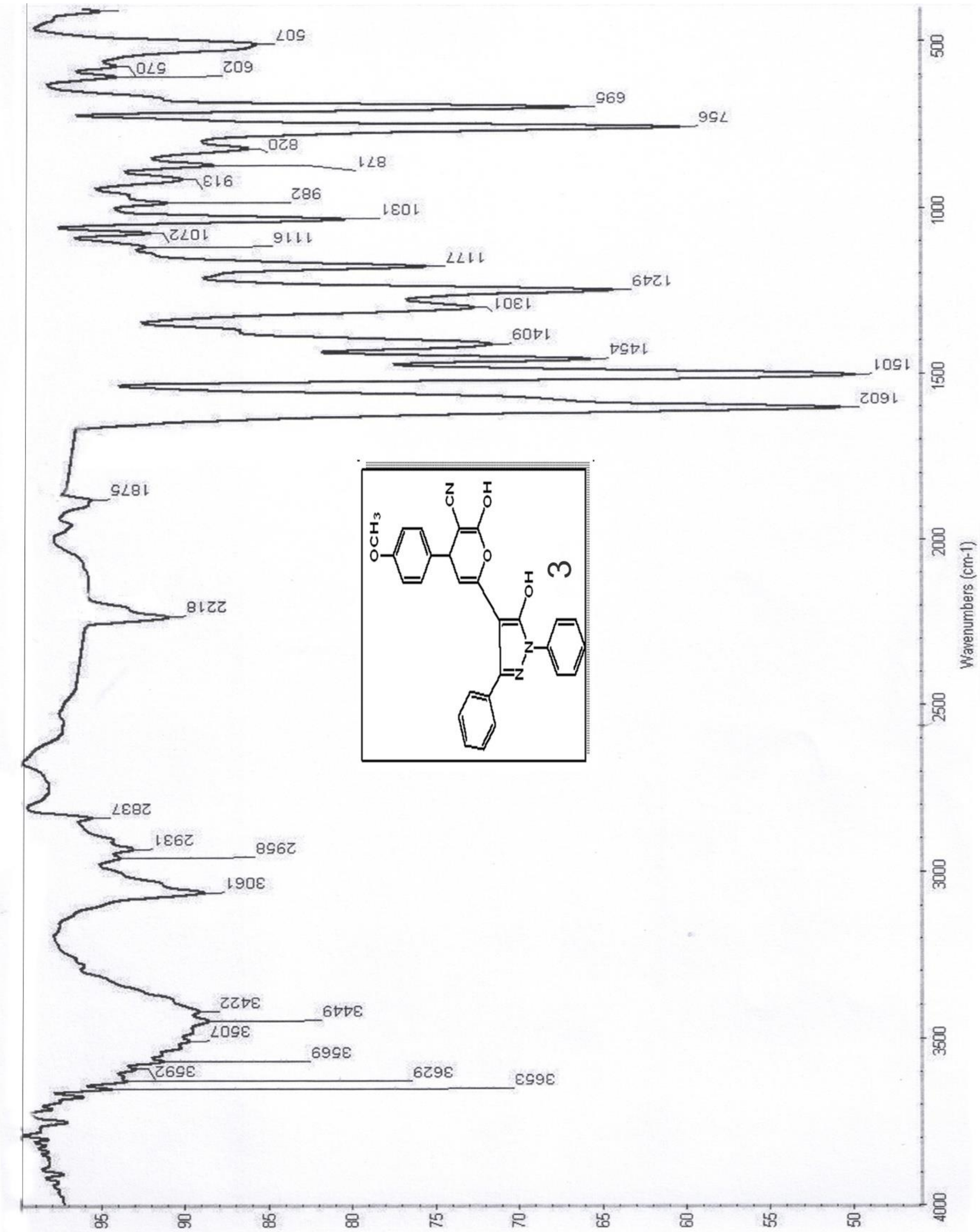

Figure S5: IR Spectrum of Compound (3)

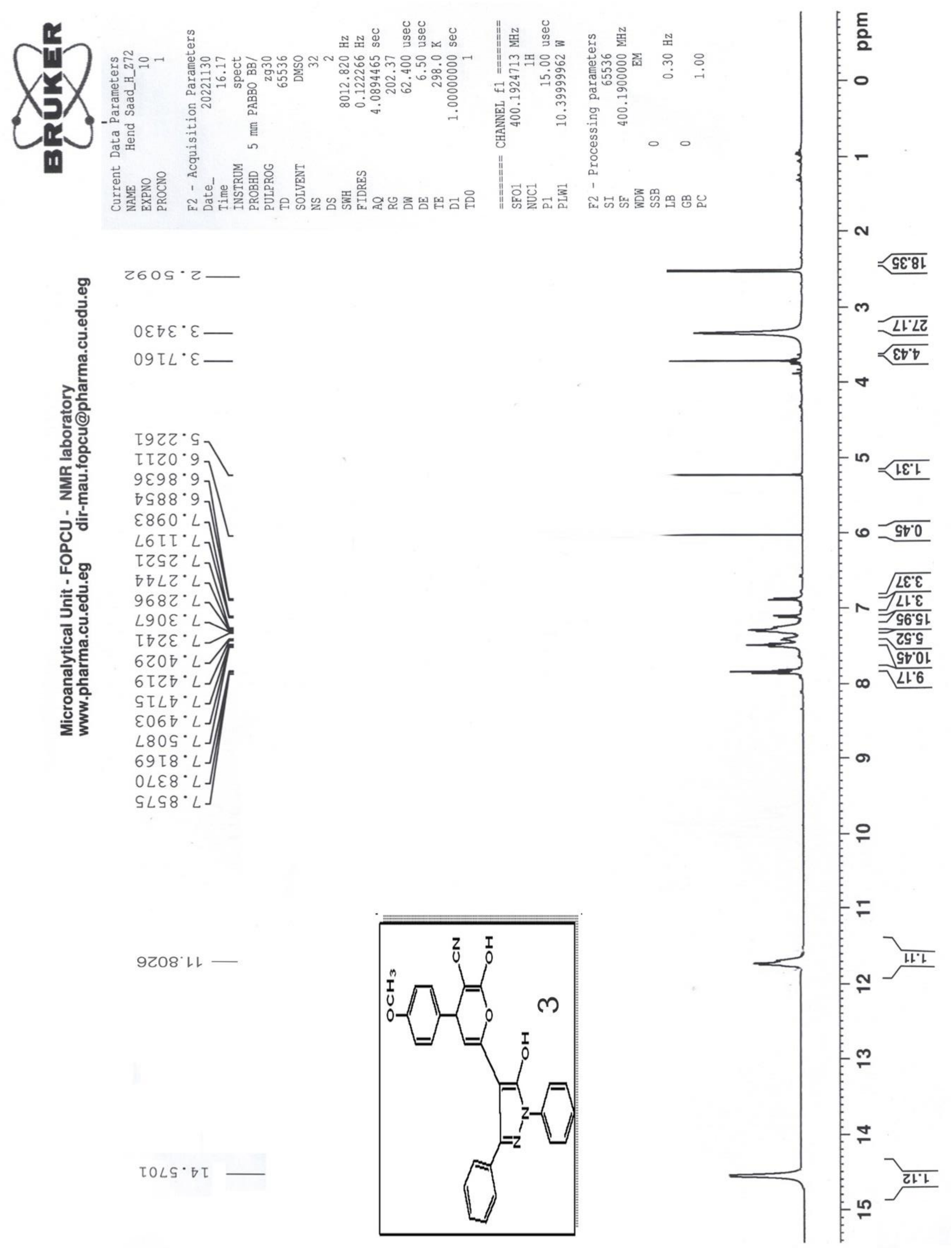

Figure S6: ^{1}H-NMR Spectrum of Compound (3) …. (DMSO)

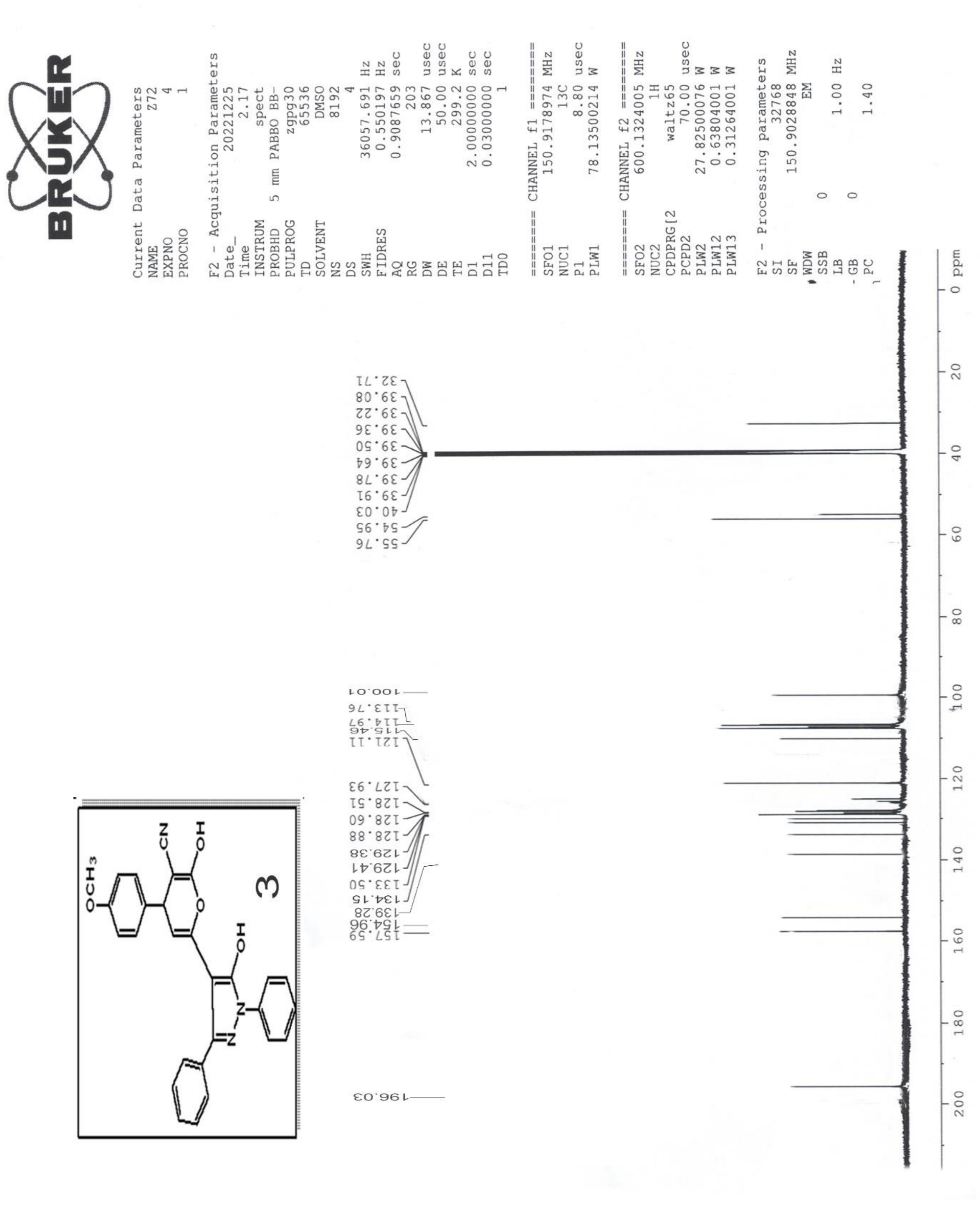

Figure S7: ^{13}C-NMR Spectrum of Compound (3) …… (DMSO)

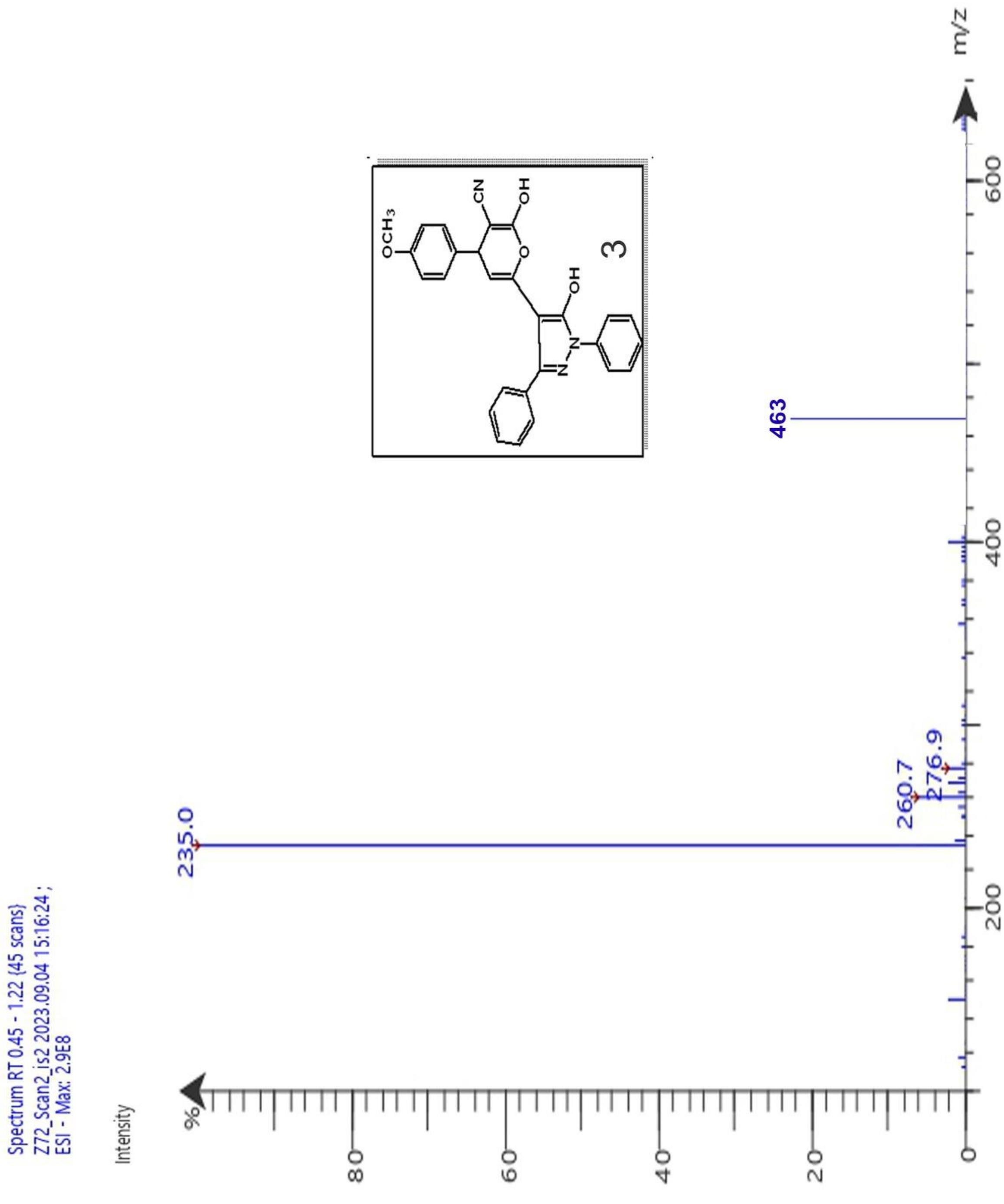

Figure S8: Mass Spectrum of Compound (3) M. wt=463 (M^+)

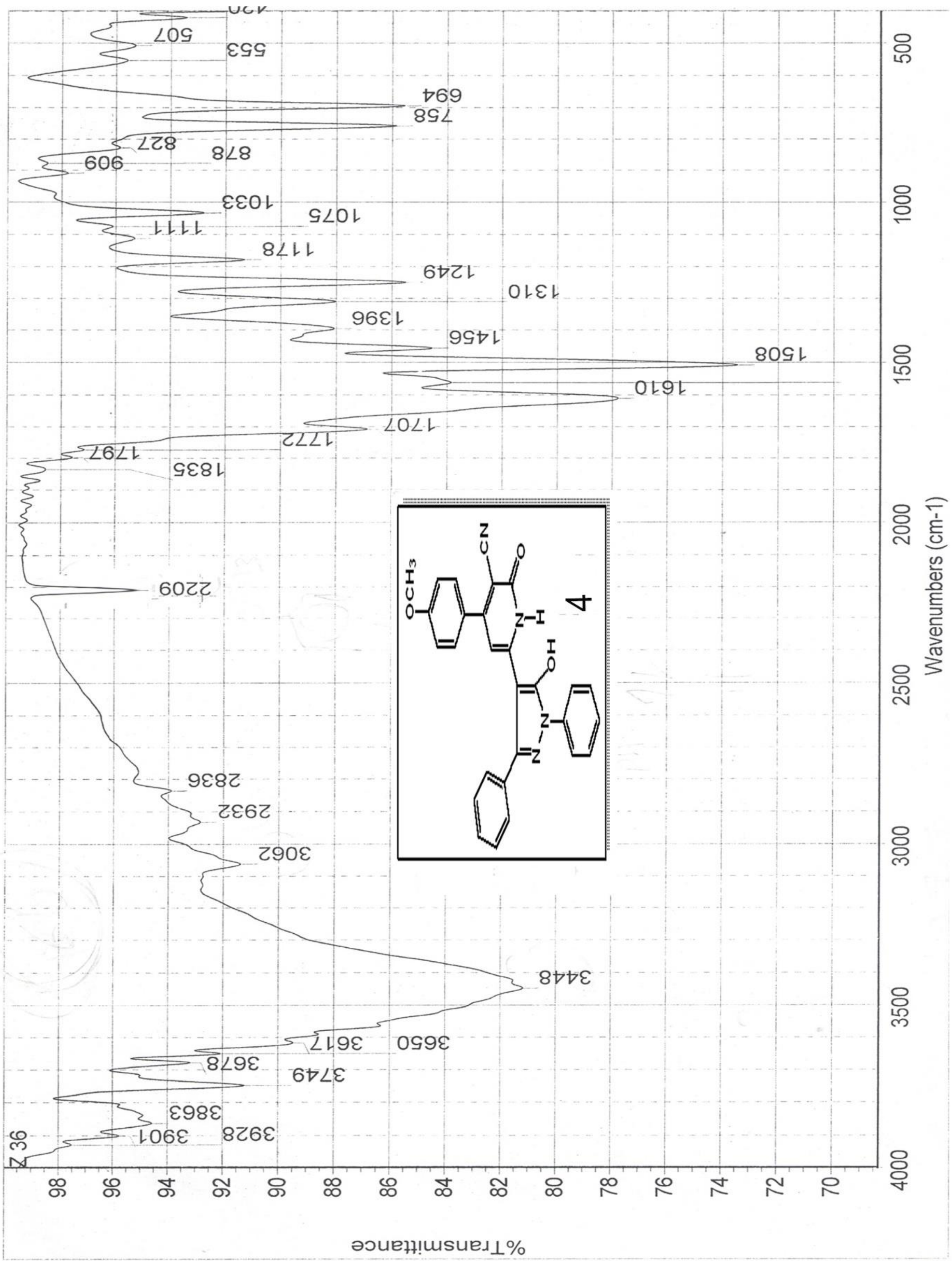

Figure S9: IR Spectrum of Compound (4)

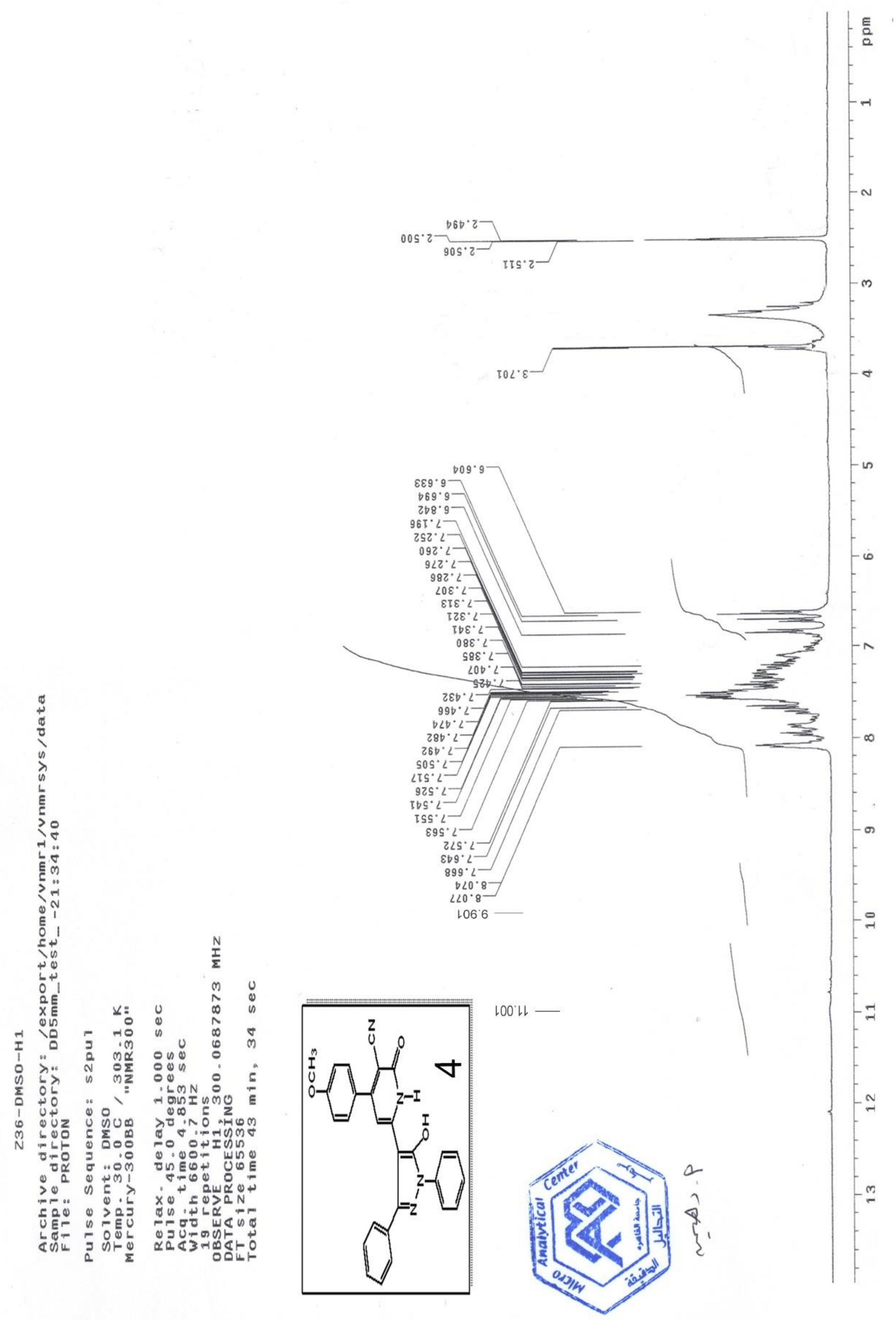

Figure S10: ^{1}H-NMR Spectrum of Compound (4) ….(DMSO)

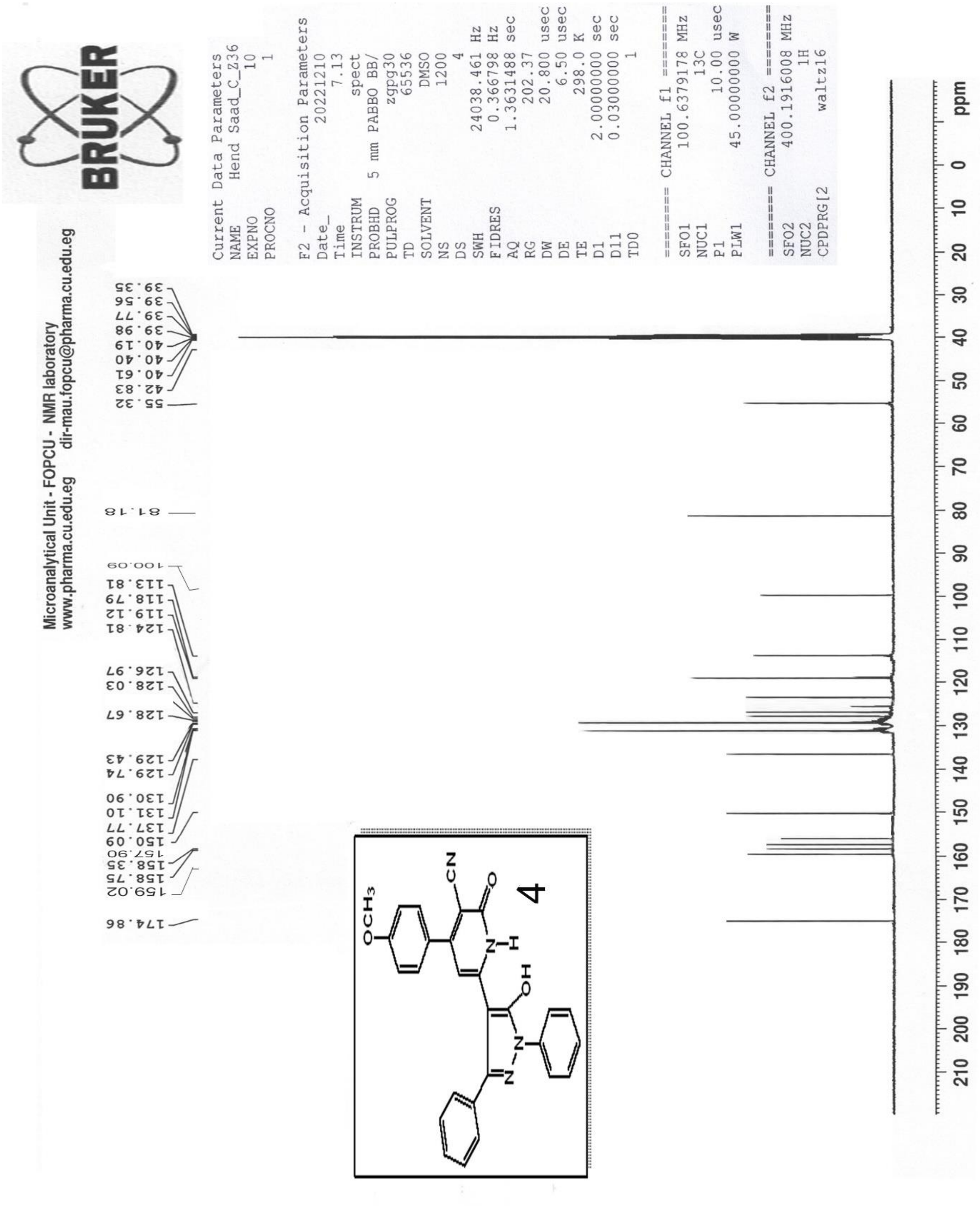

Figure S11: ^{13}C-NMR Spectrum of Compound (4) …… (DMSO)

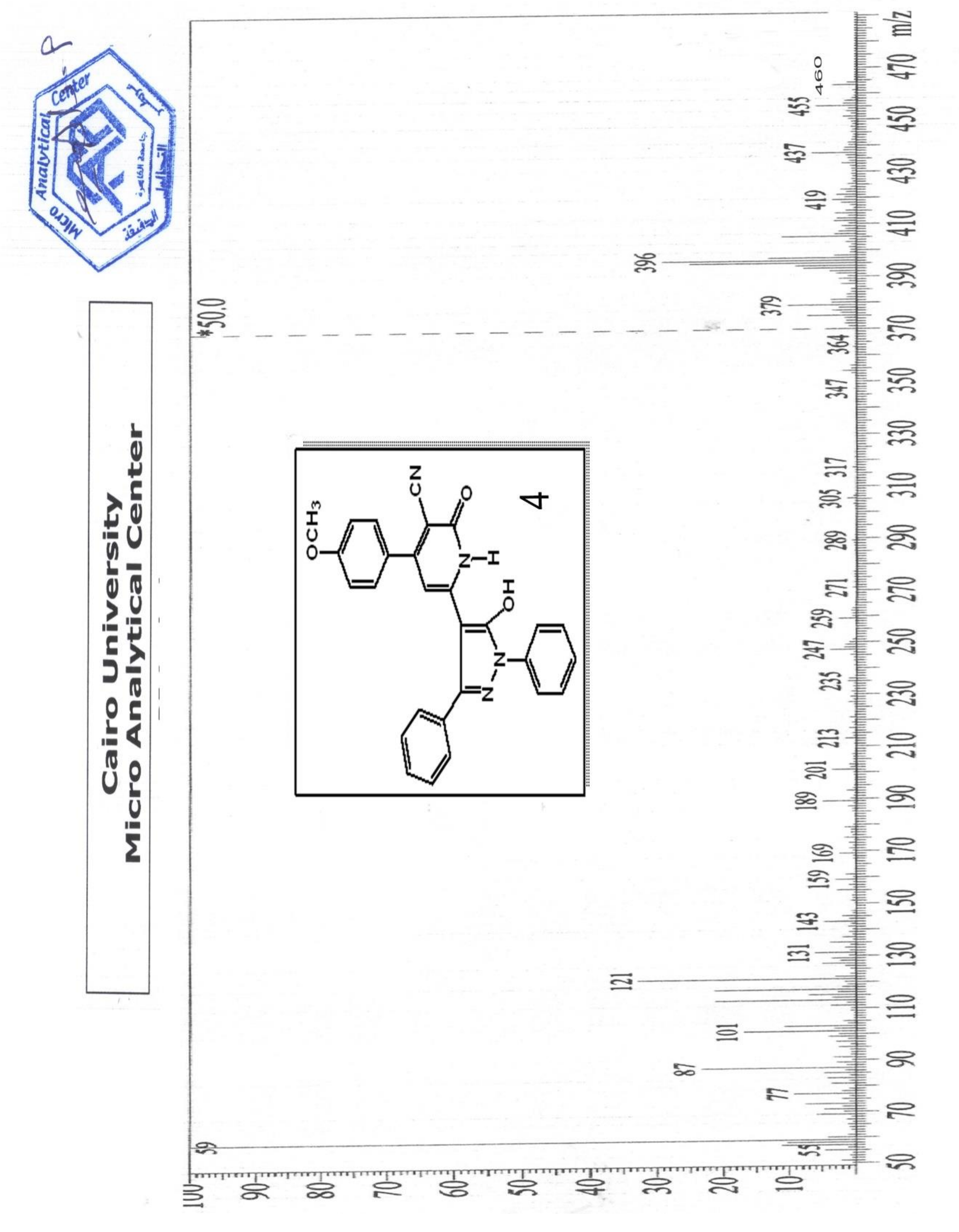

Figure S12: Mass Spectrum of Compound (4) M.wt=460 (M^+)

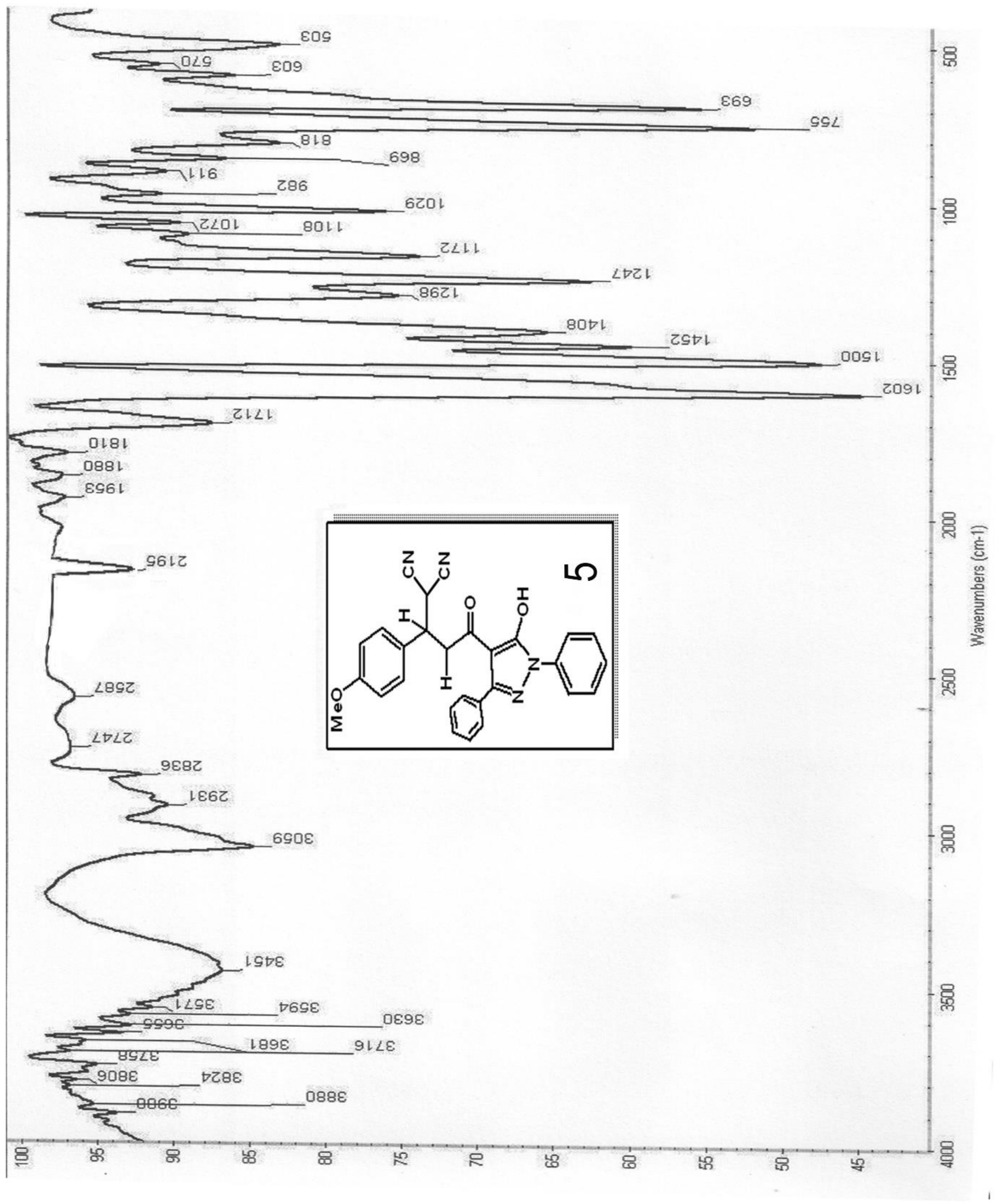

Figure S13: IR Spectrum of Compound (5)

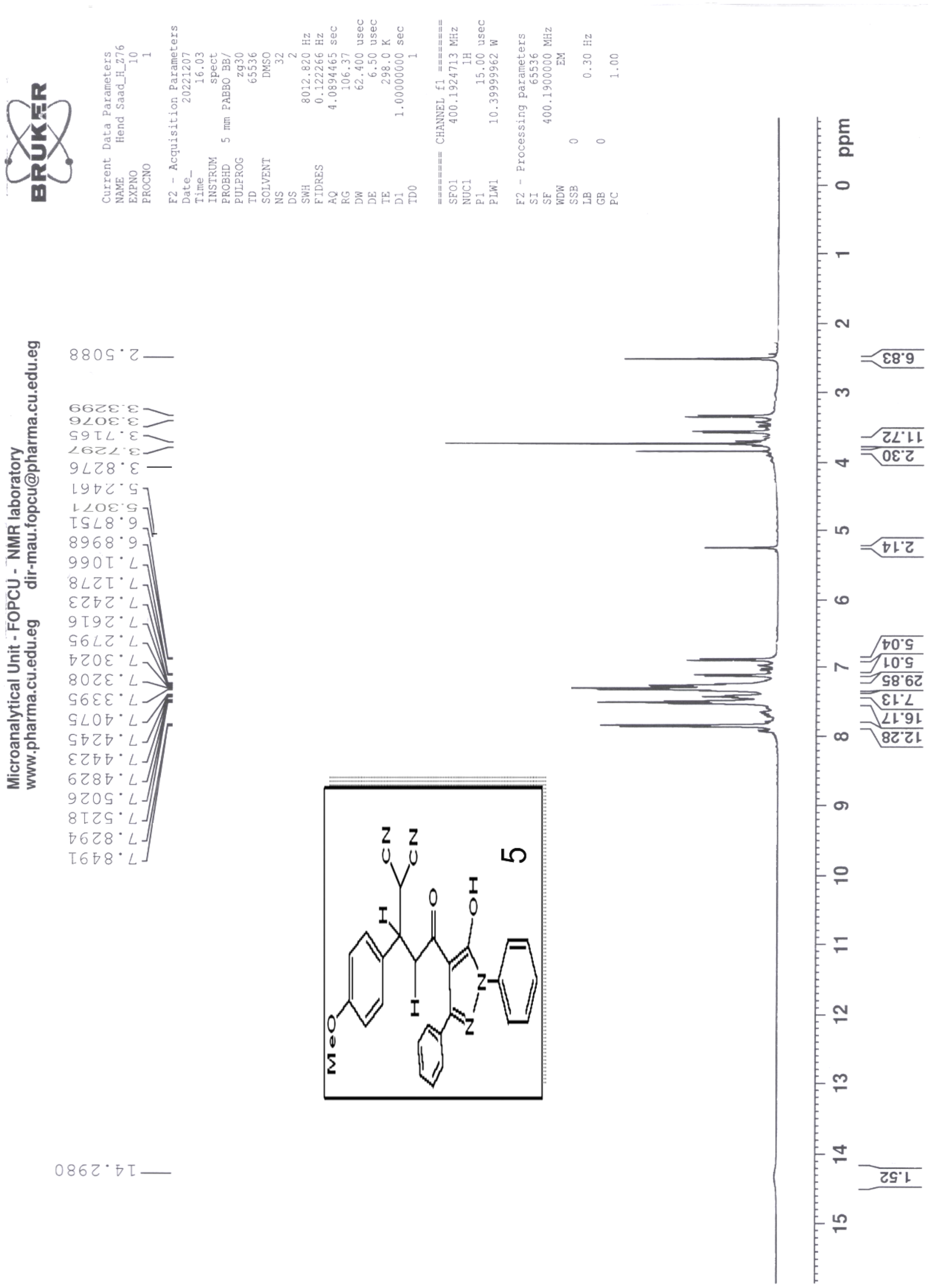

Figure S14: ^{1}H-NMR Spectrum of Compound (5) …. (DMSO)

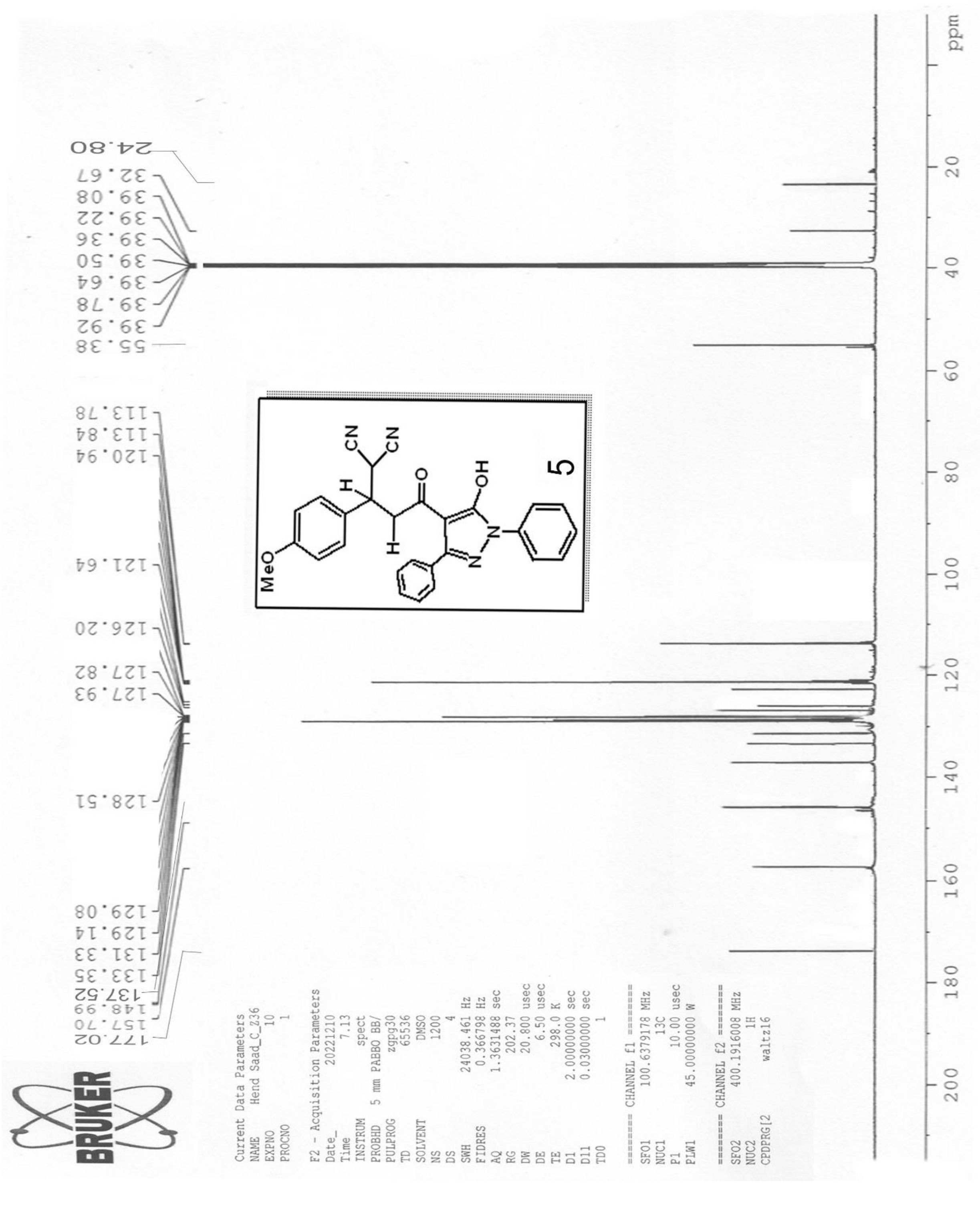

Figure S15: ^{13}C-NMR Spectrum of Compound (5) …… (DMSO)

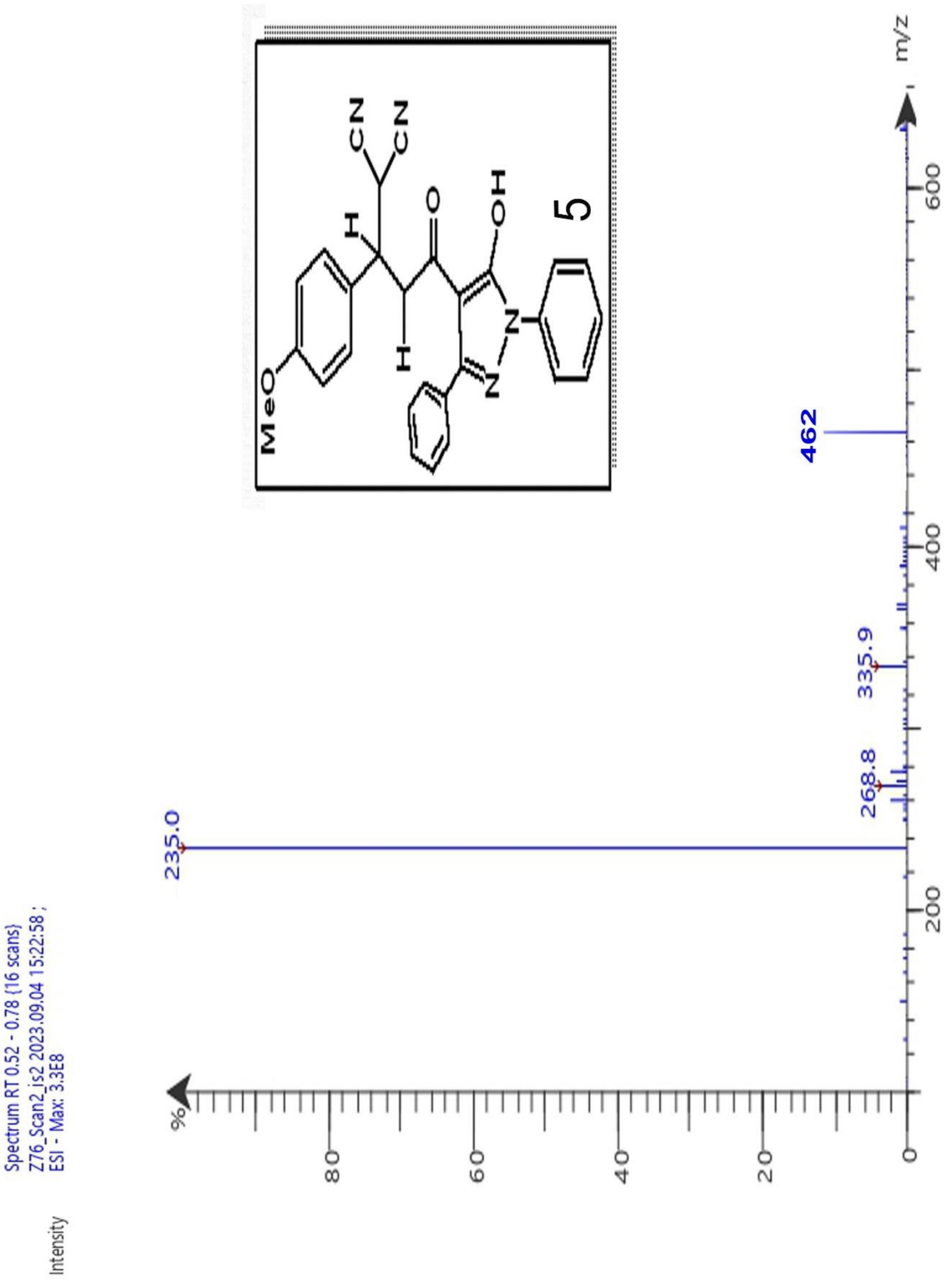

Figure S16: Mass Spectrum of Compound (5) M.wt=462 (M^+)

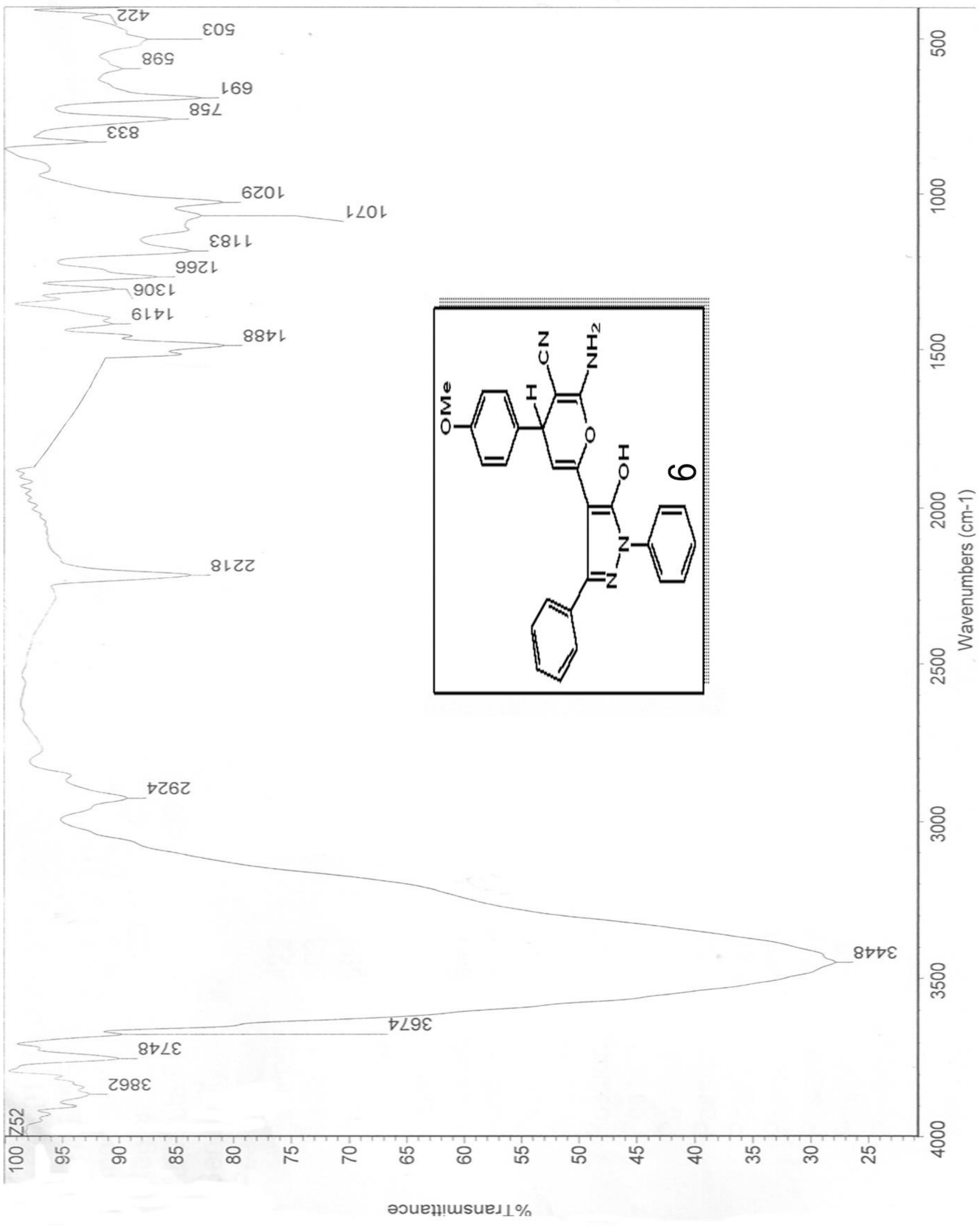

Figure S17: IR Spectrum of Compound (6)

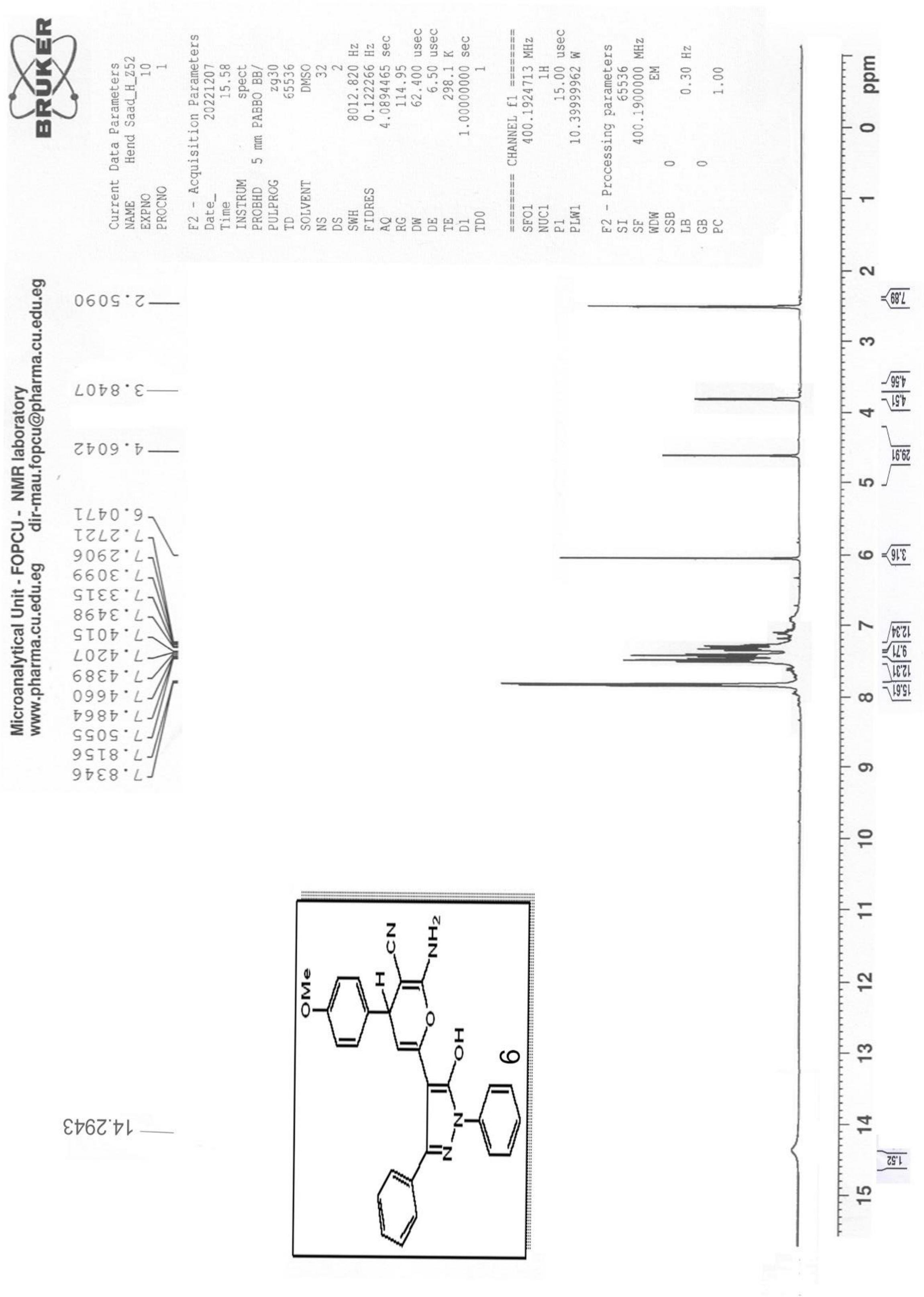

Figure S18: ^{1}H-NMR Spectrum of Compound (6) …. (DMSO)

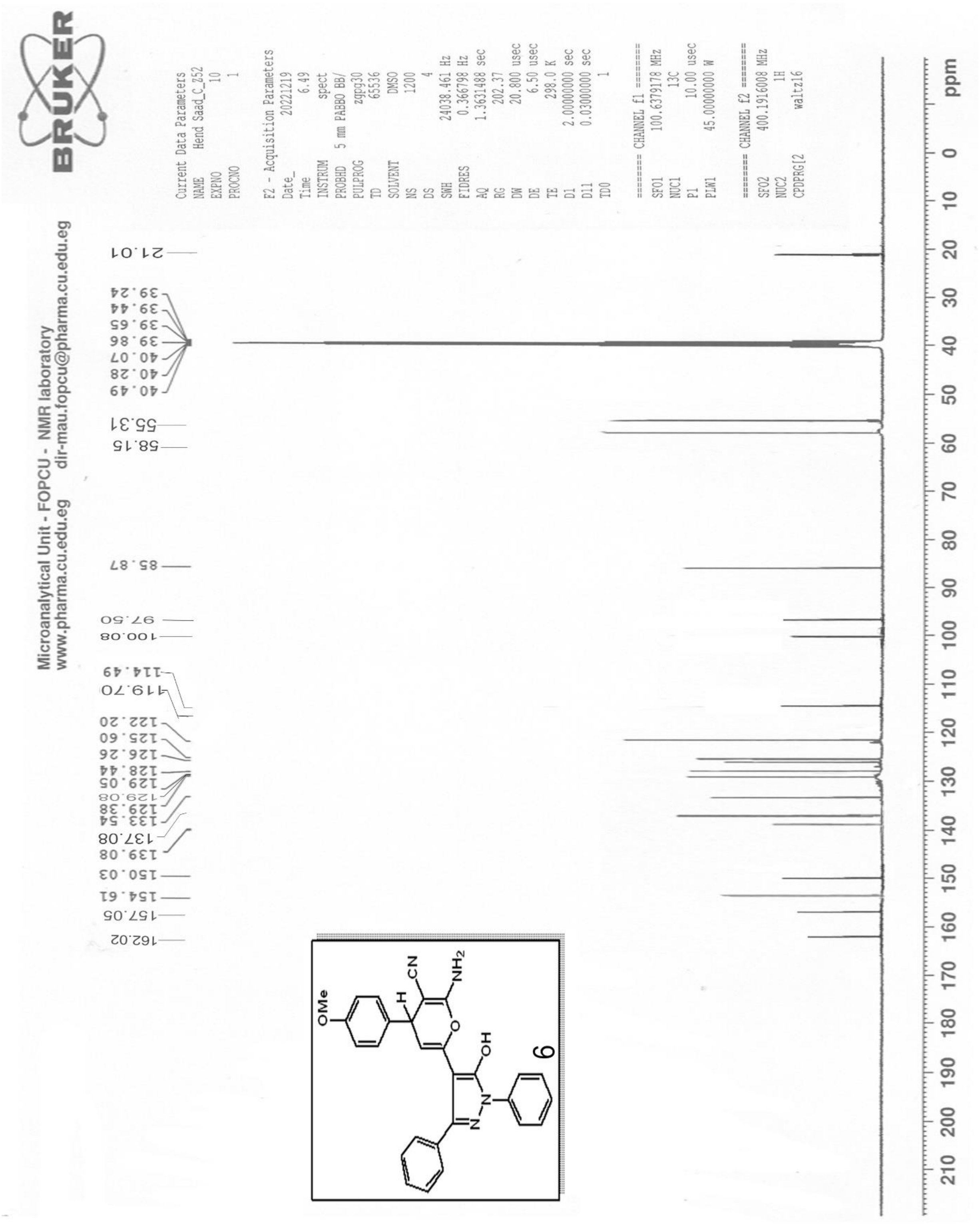

Figure S19: ^{13}C-NMR Spectrum of Compound (6) …… (DMSO)

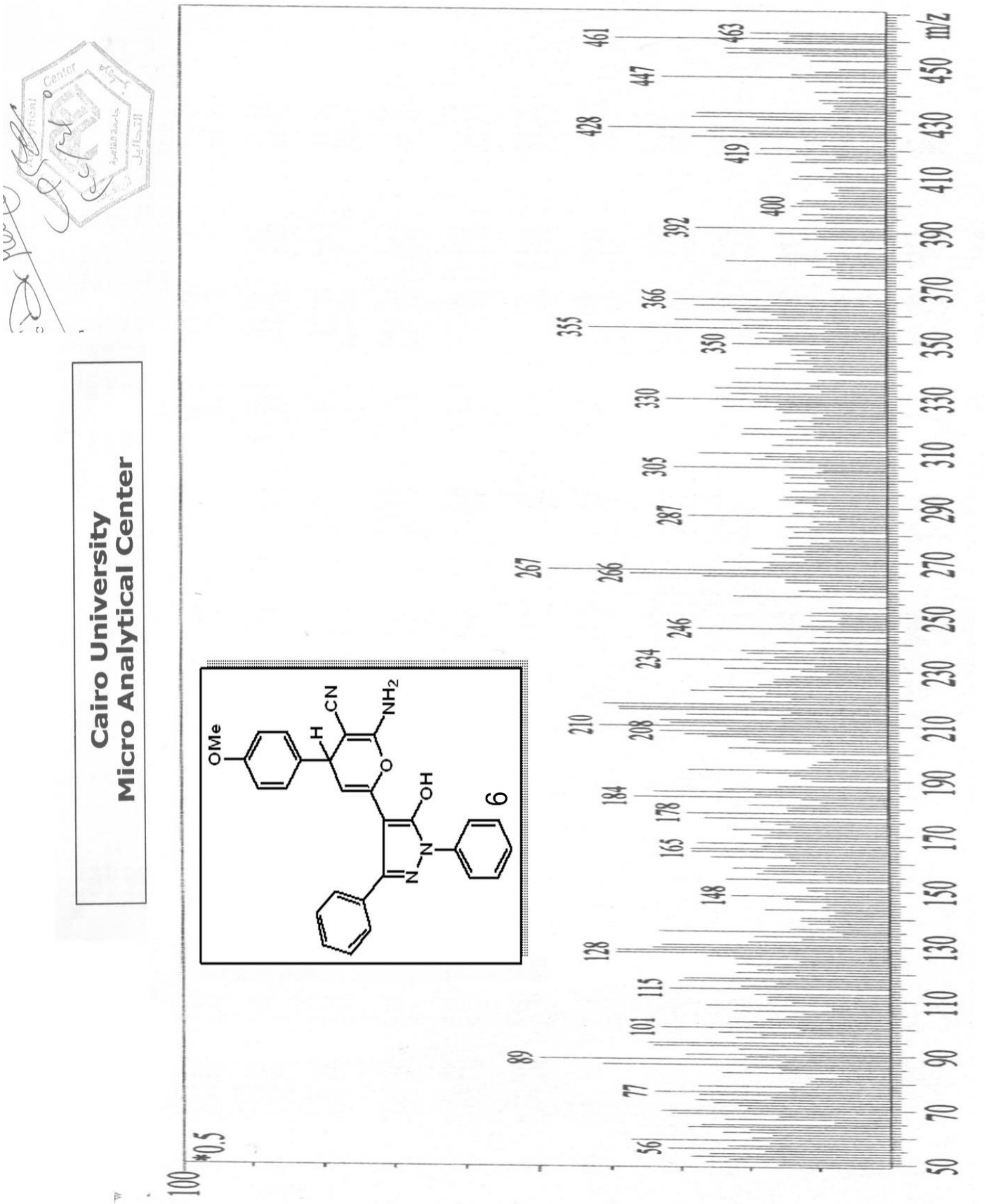

Figure S20: Mass Spectrum of Compound (7) M.wt=463 (M^{+}+1)

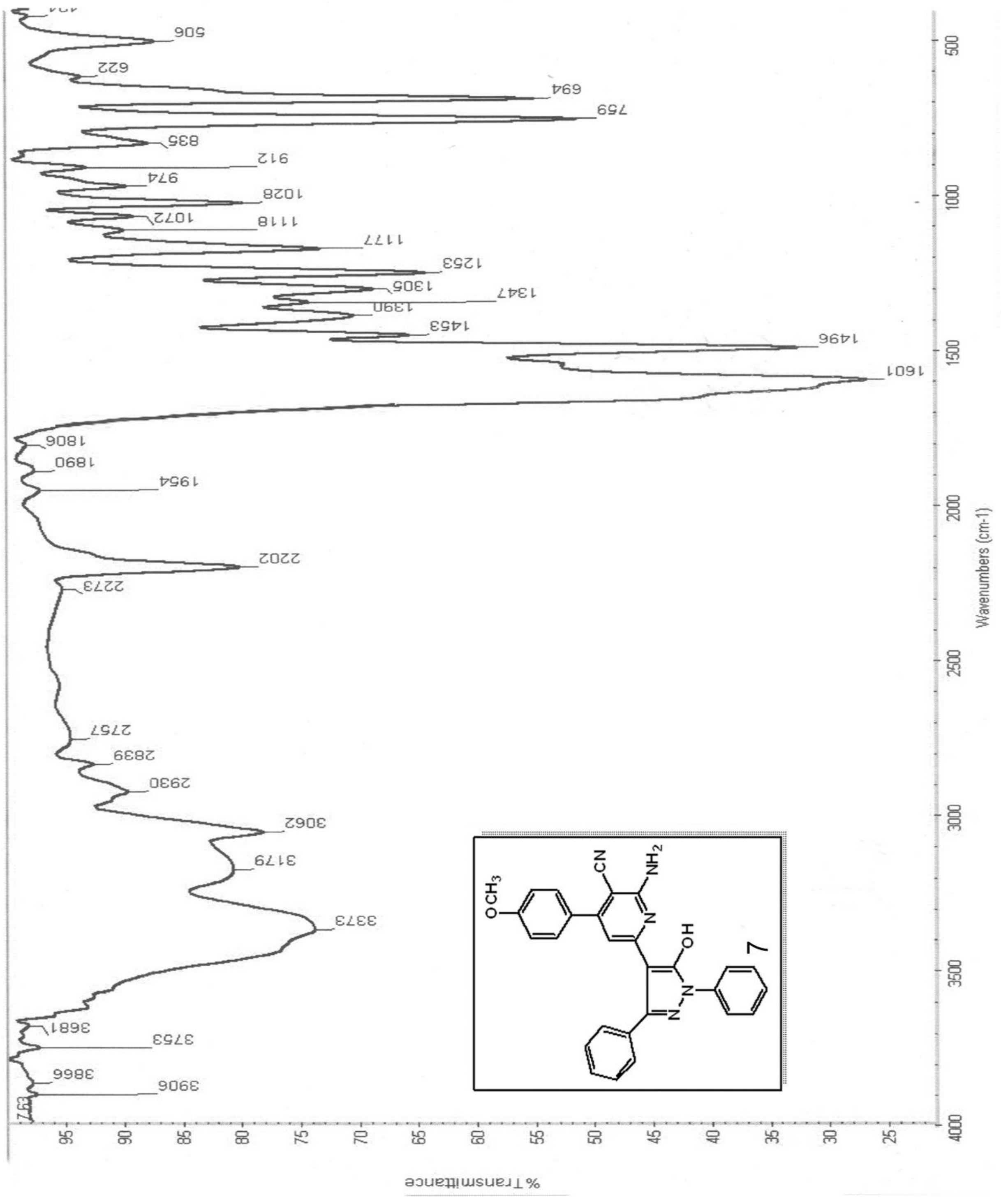

Figure S21: IR Spectrum of Compound (7)

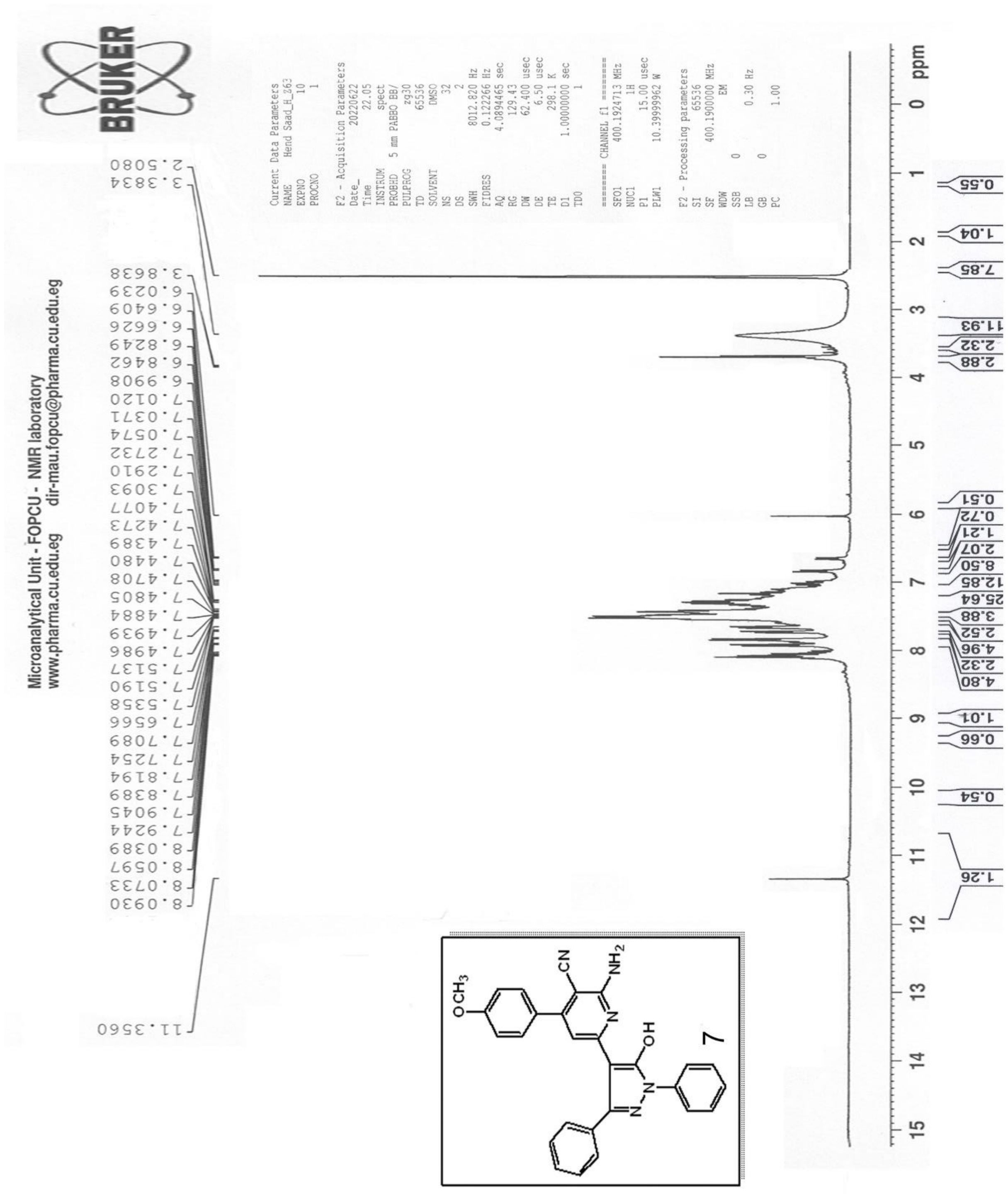

Figure S22: ^{1}H-NMR Spectrum of Compound (7) …. (DMSO)

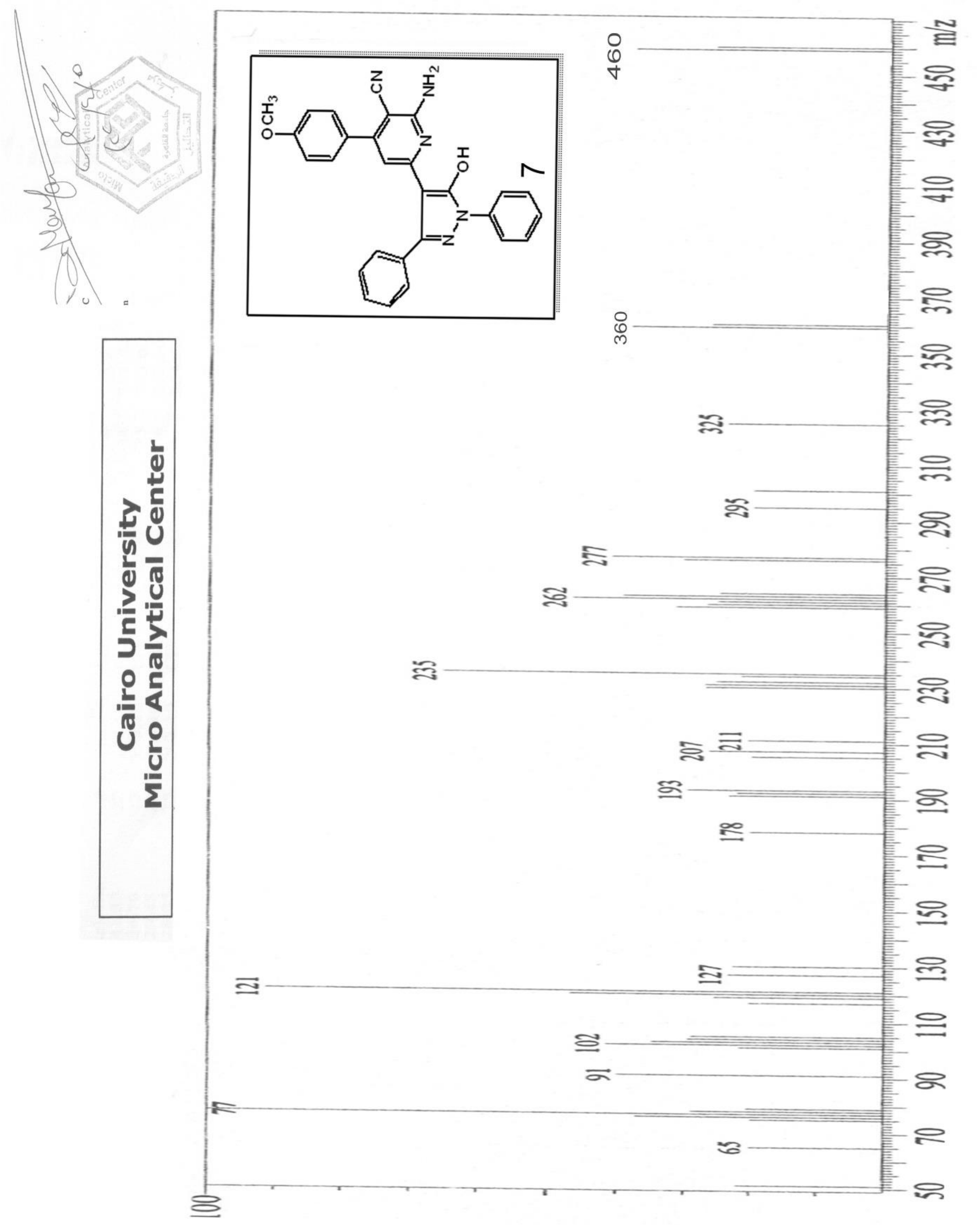

Figure S23: Mass Spectrum of Compound (7) M.wt=460 (M^{+}+1)

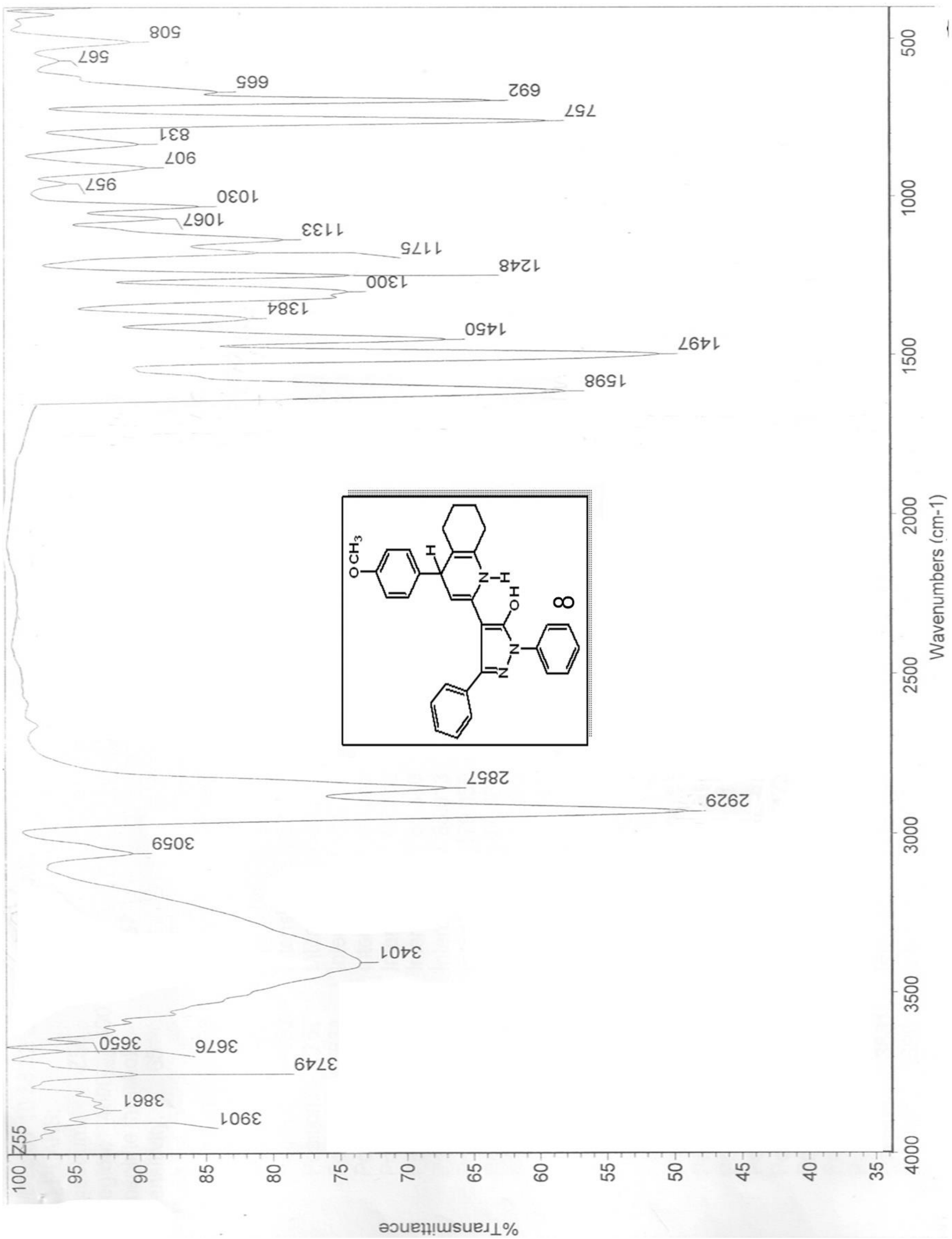

Figure S24: IR Spectrum of Compound (8)

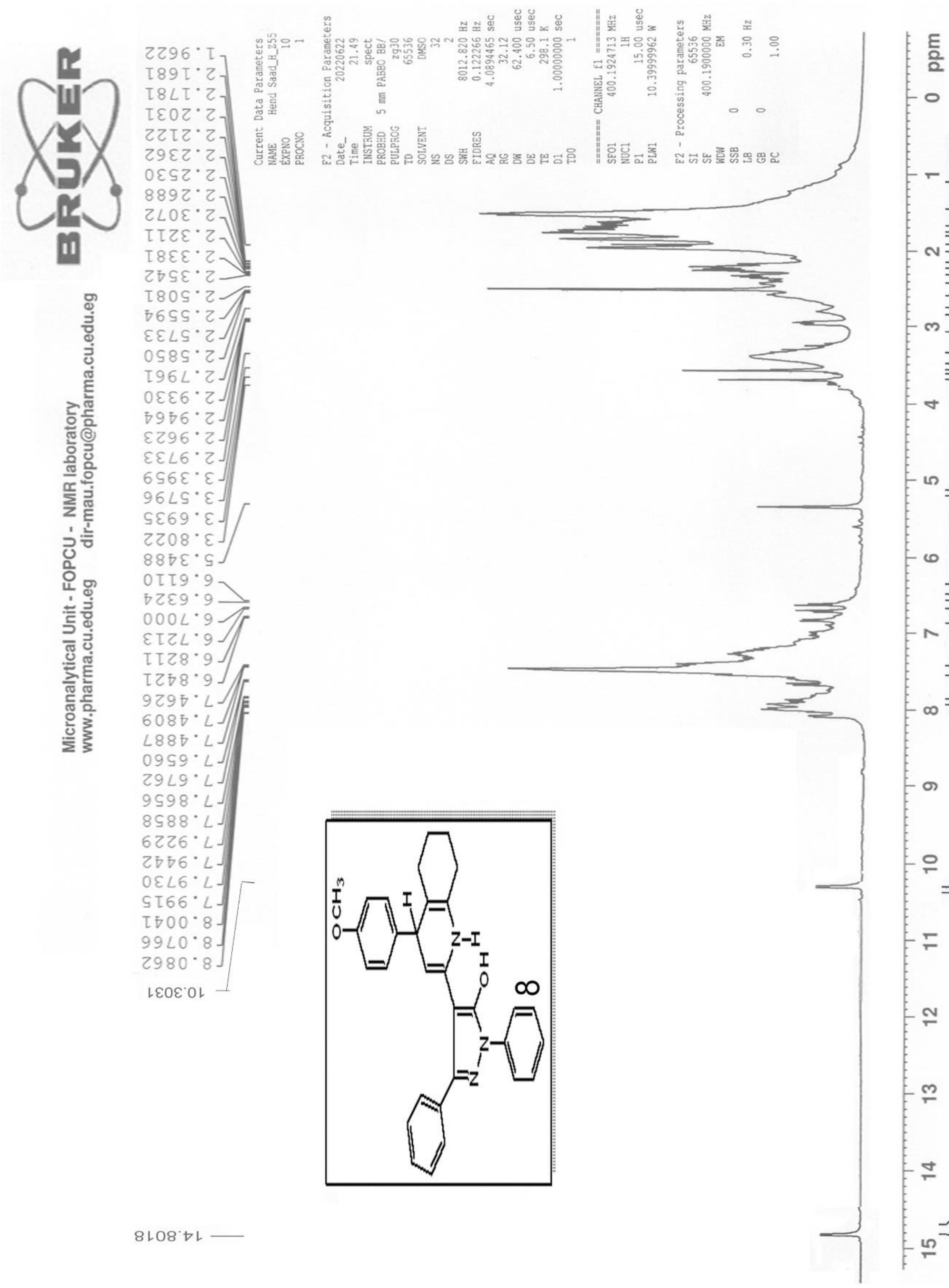

Figure S25: ^{1}H-NMR Spectrum of Compound (8) …. (DMSO)

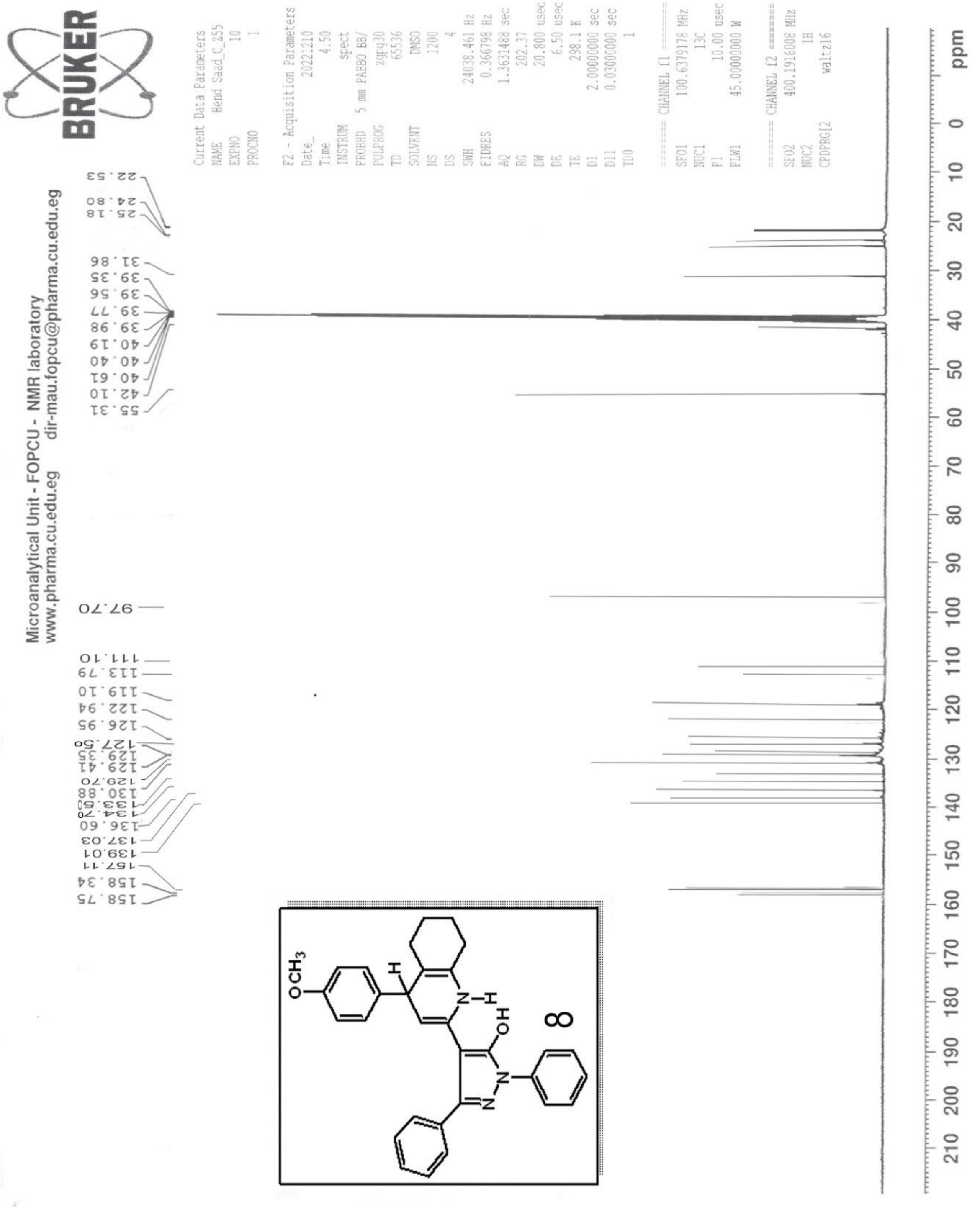

Figure S26: ^{13}C-NMR Spectrum of Compound (8) …… (DMSO)

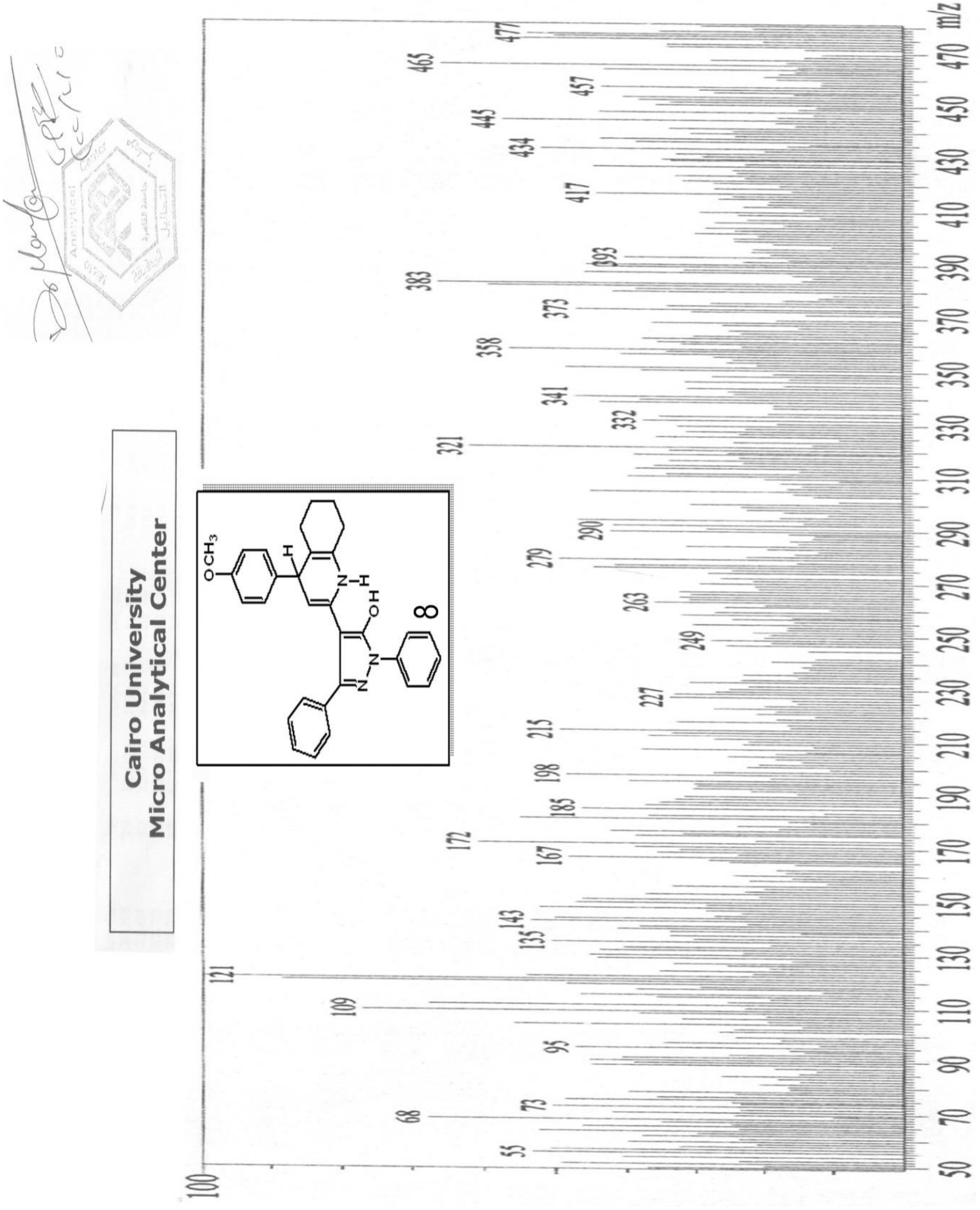

Figure S27: Mass Spectrum of Compound (8) M.wt=477 (M^++2)

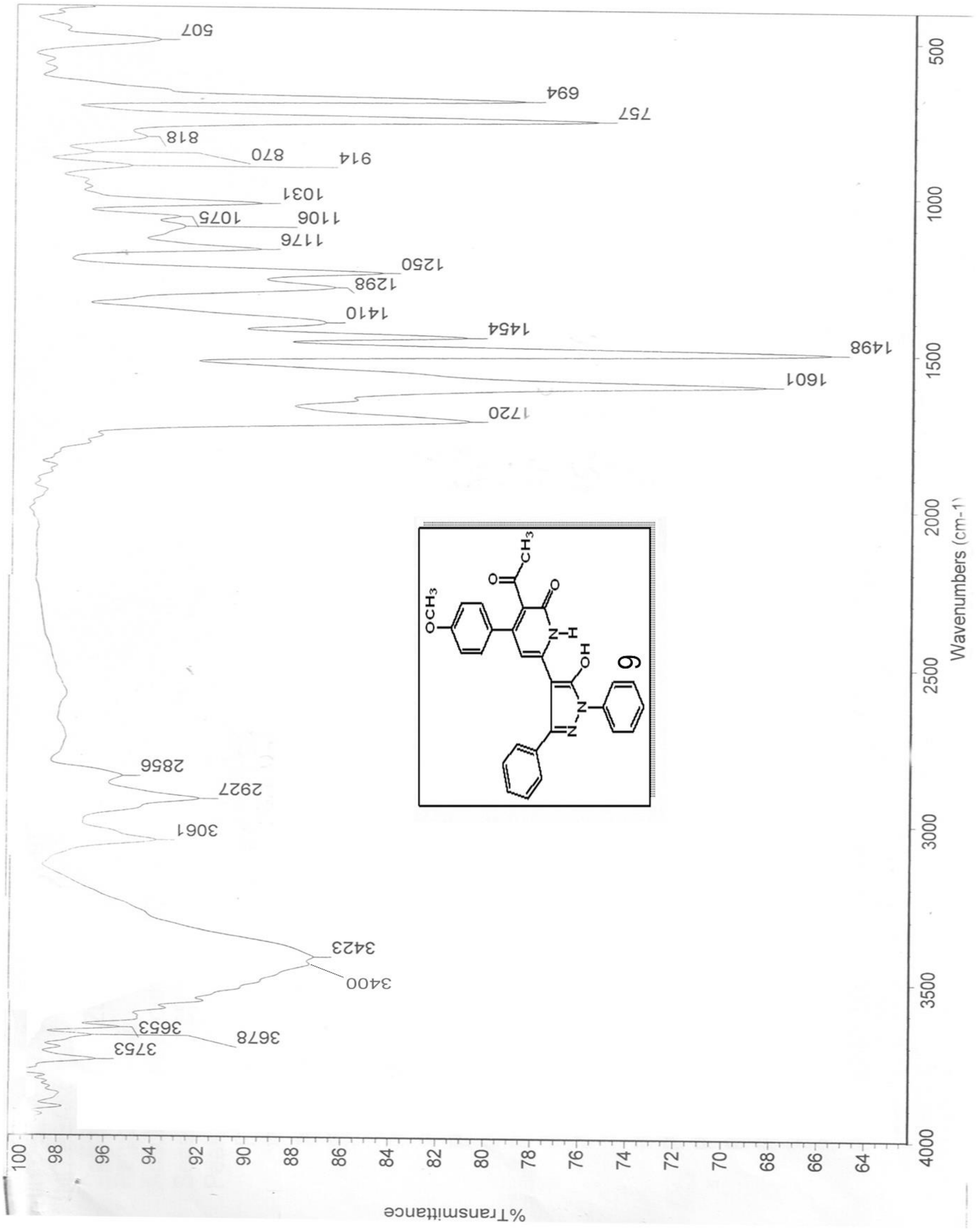

Figure S28: IR Spectrum of Compound (9)

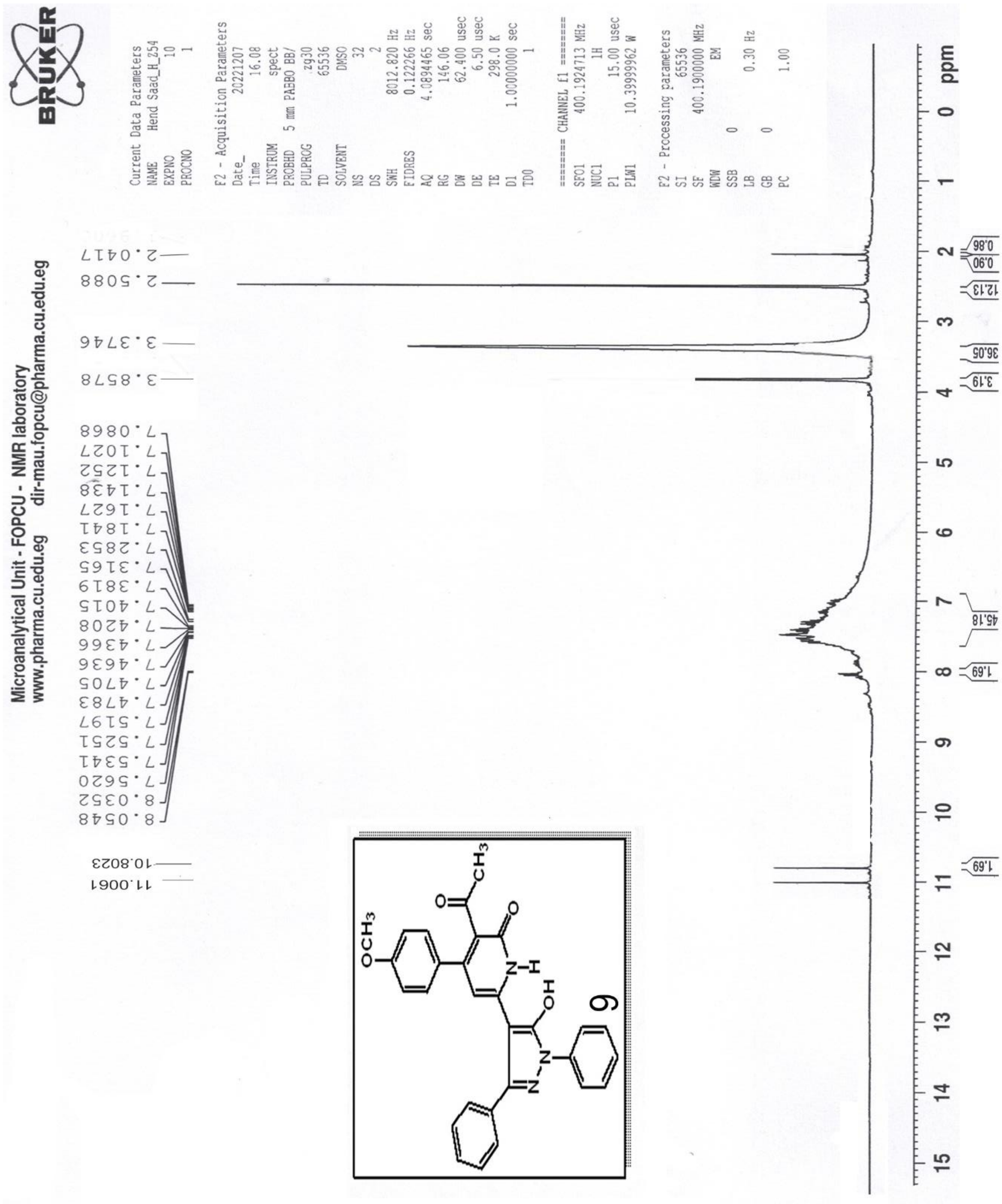

Figure S29: ^{1}H-NMR Spectrum of Compound (9) …. (DMSO)

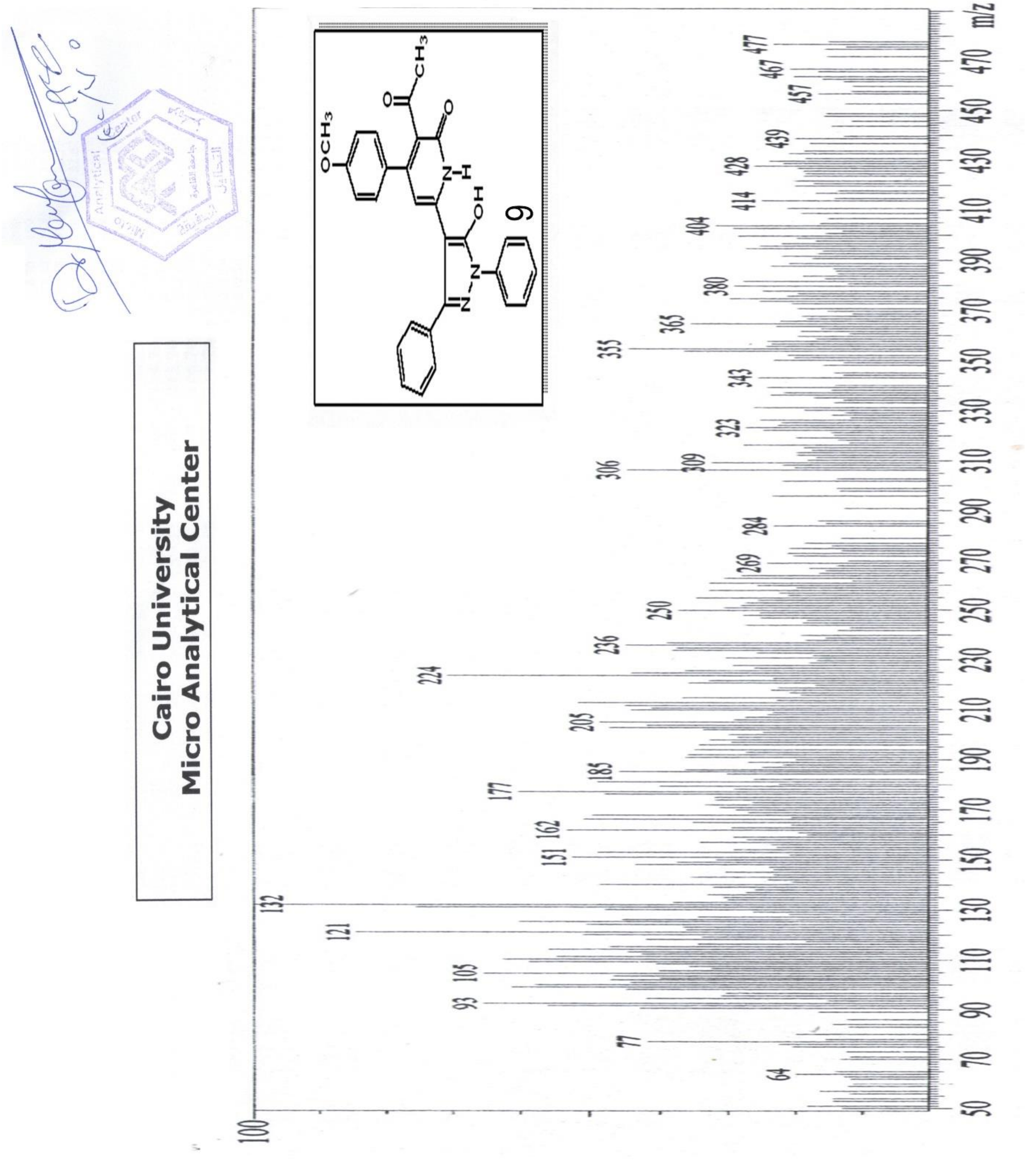

Figure S30: Mass Spectrum of Compound (9) M.wt=477 (M^+)

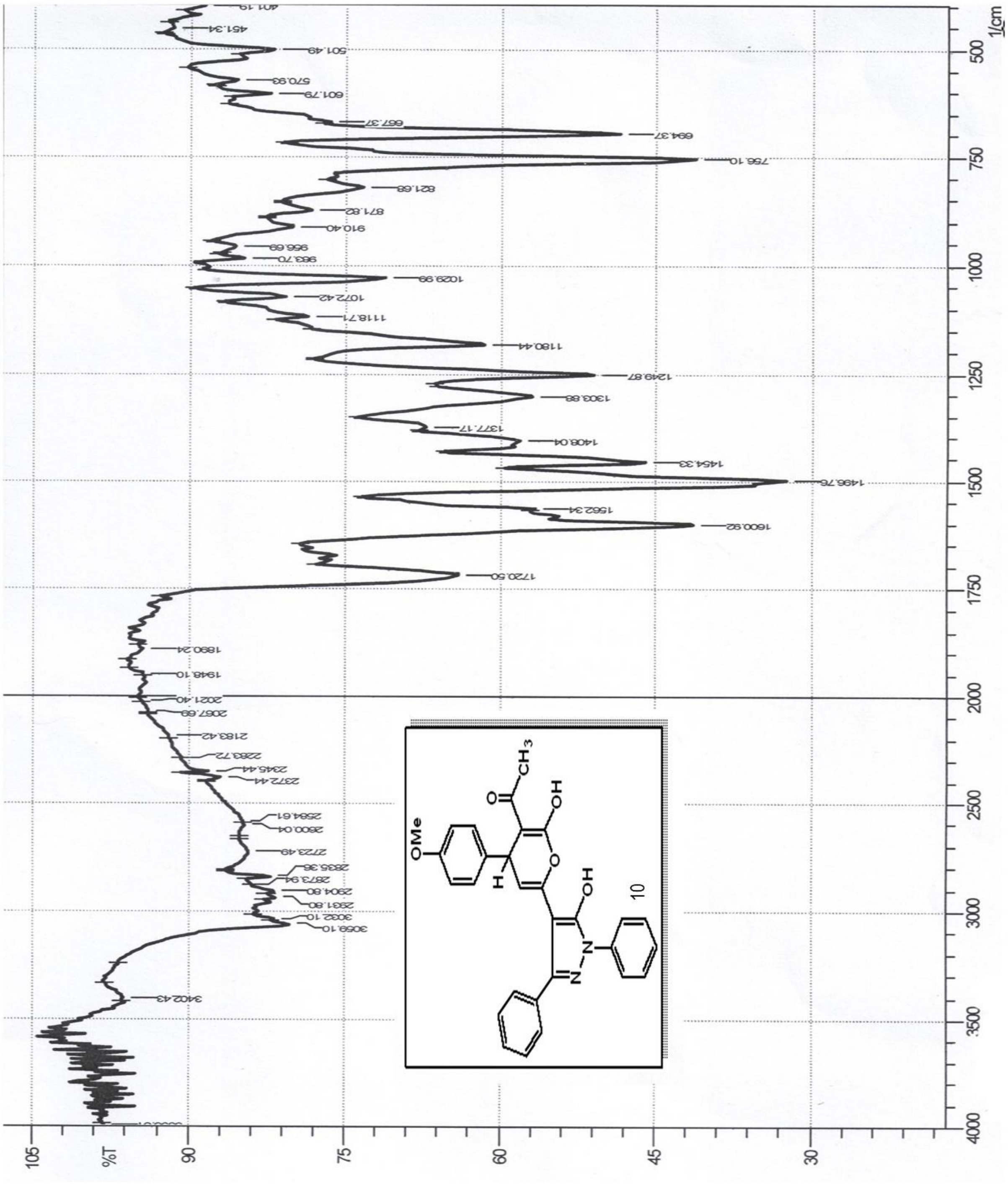

Figure S31: IR Spectrum of Compound (10)

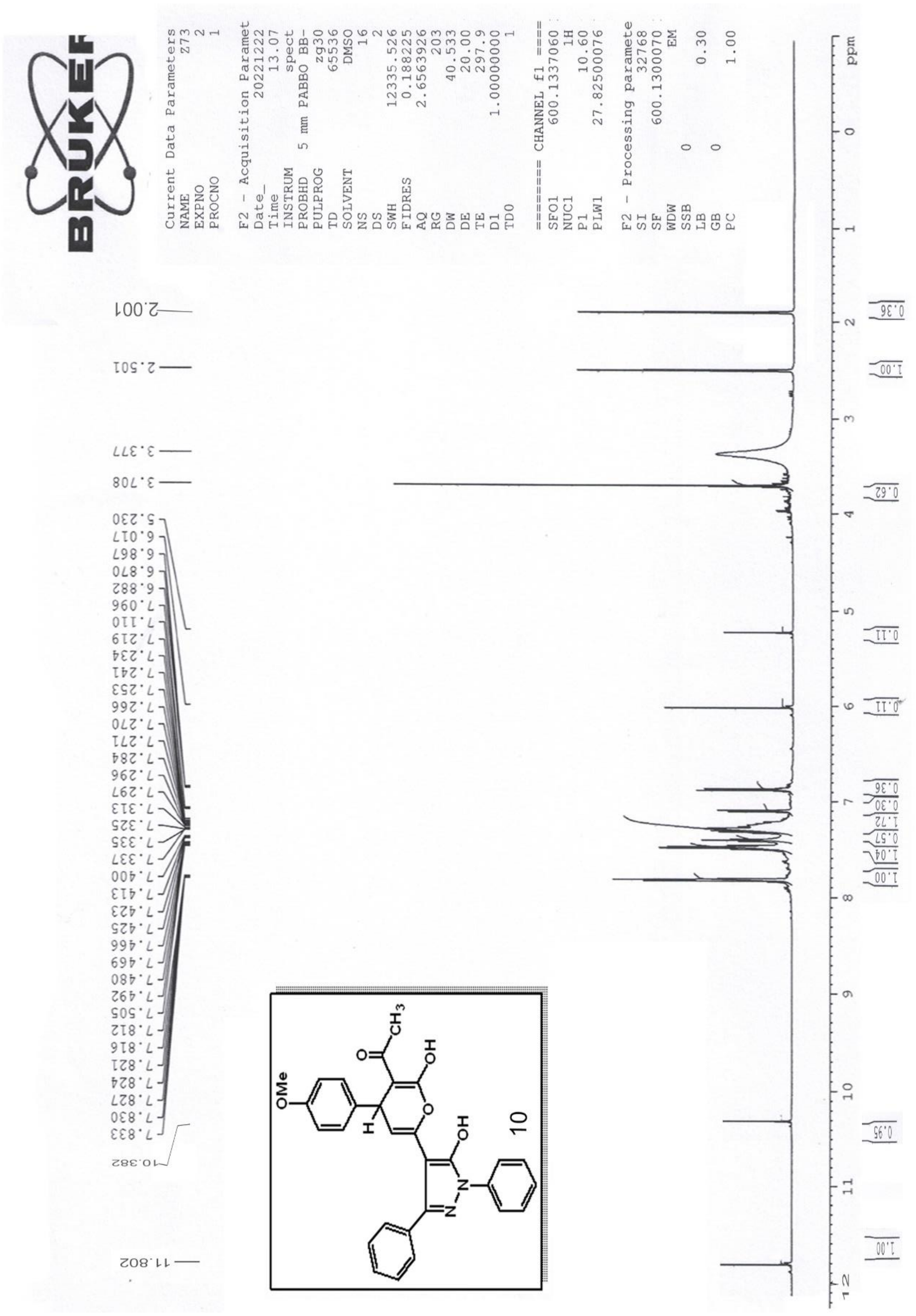

Figure S32: ^{1}H-NMR Spectrum of Compound (10) …. (DMSO)

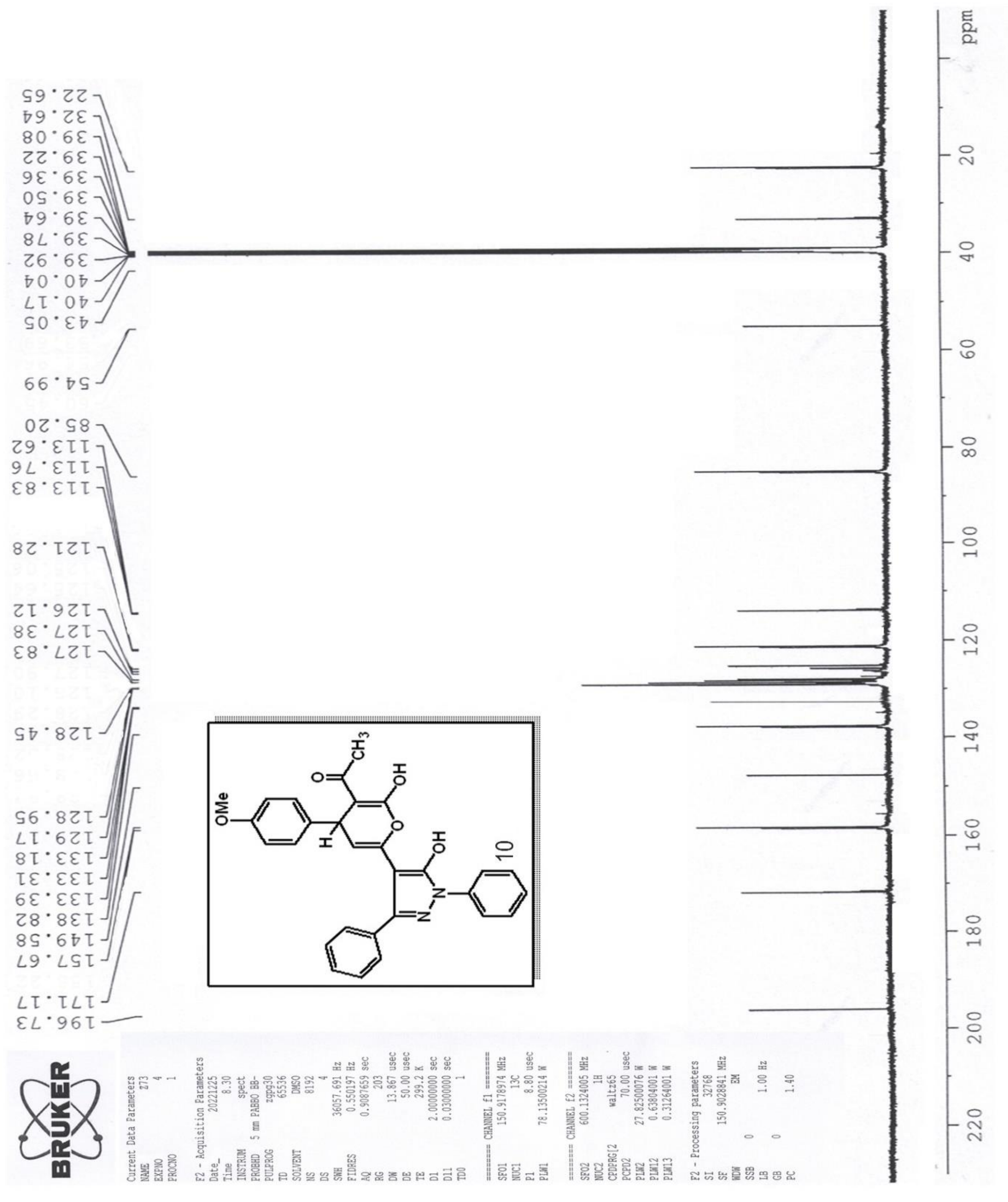

Figure S33: ^{13}C-NMR Spectrum of Compound (10) …… (DMSO)

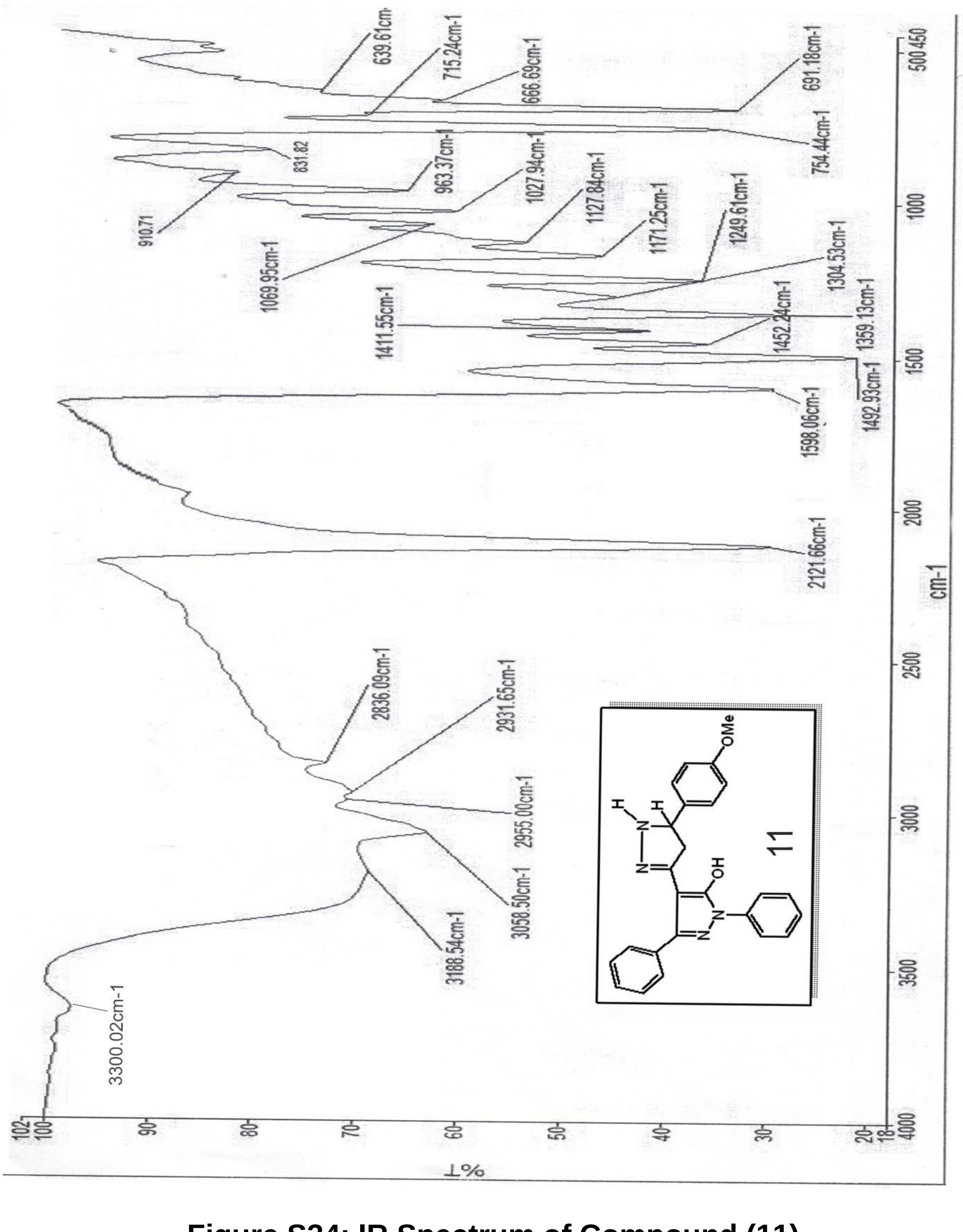

Figure S34: IR Spectrum of Compound (11)

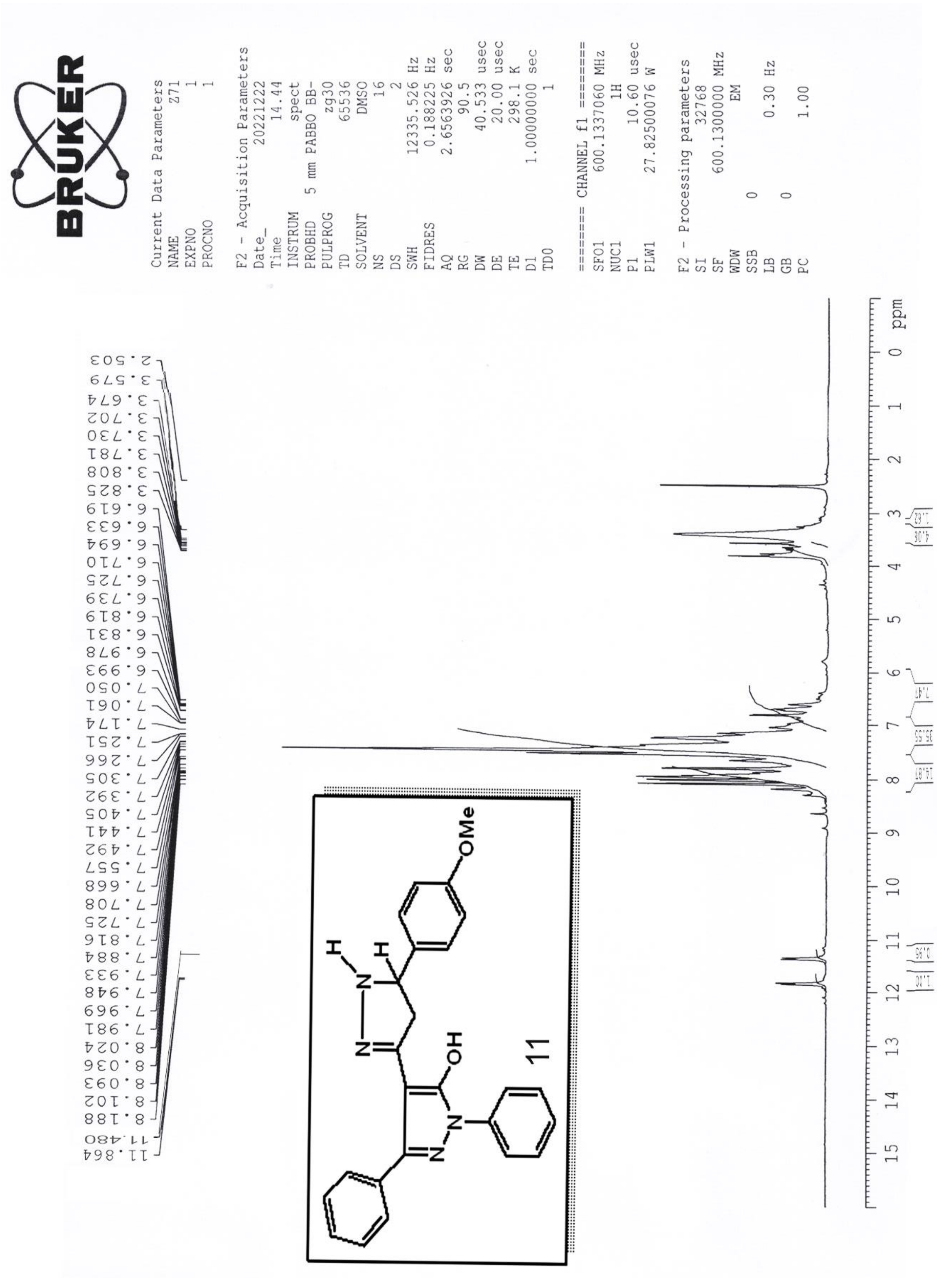

Figure S35: ^{1}H-NMR Spectrum of Compound (11)….(DMSO)

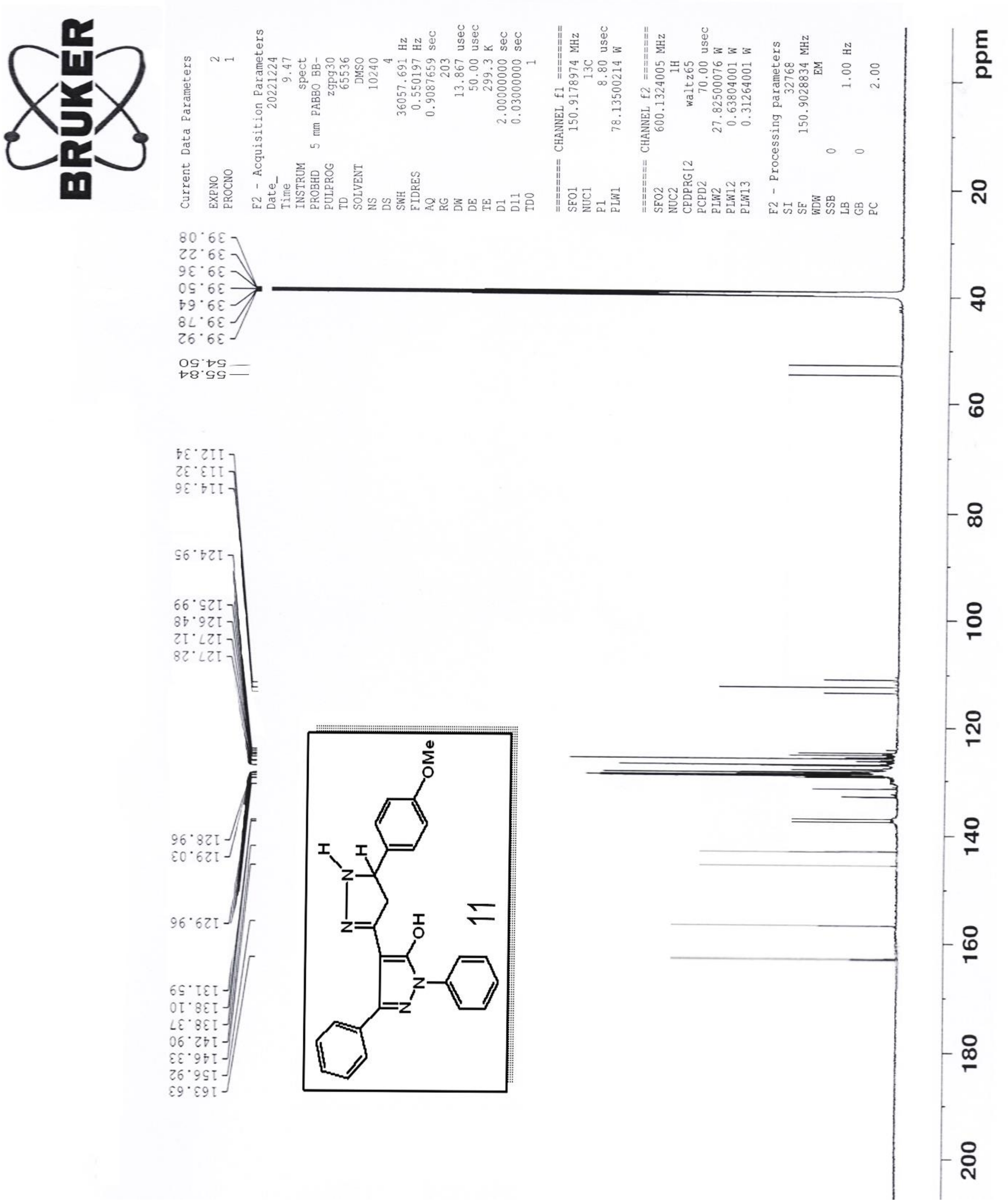

Figure S36: ^{13}C-NMR Spectrum of Compound (11) …… (DMSO)

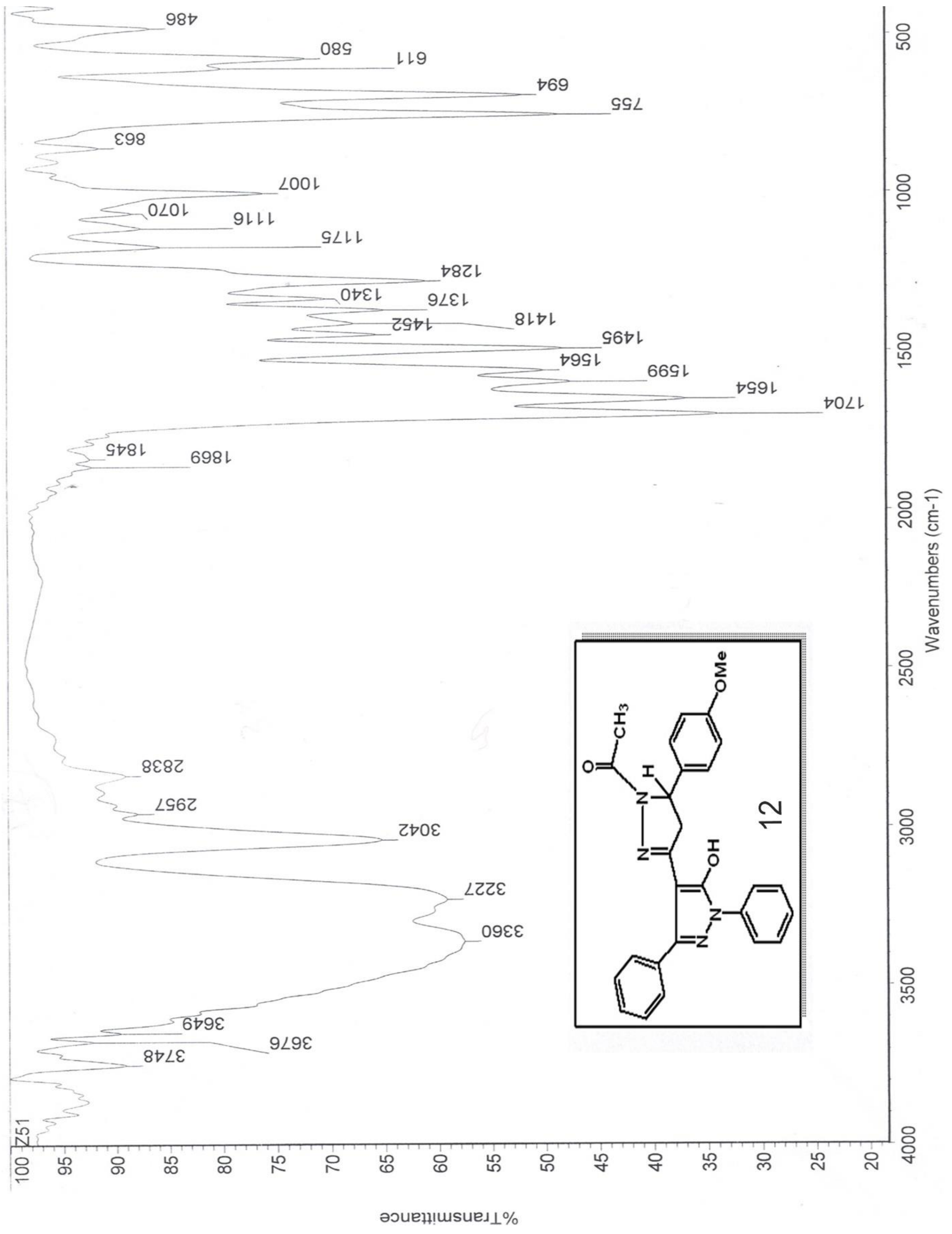

Figure S37: IR Spectrum of Compound (12)

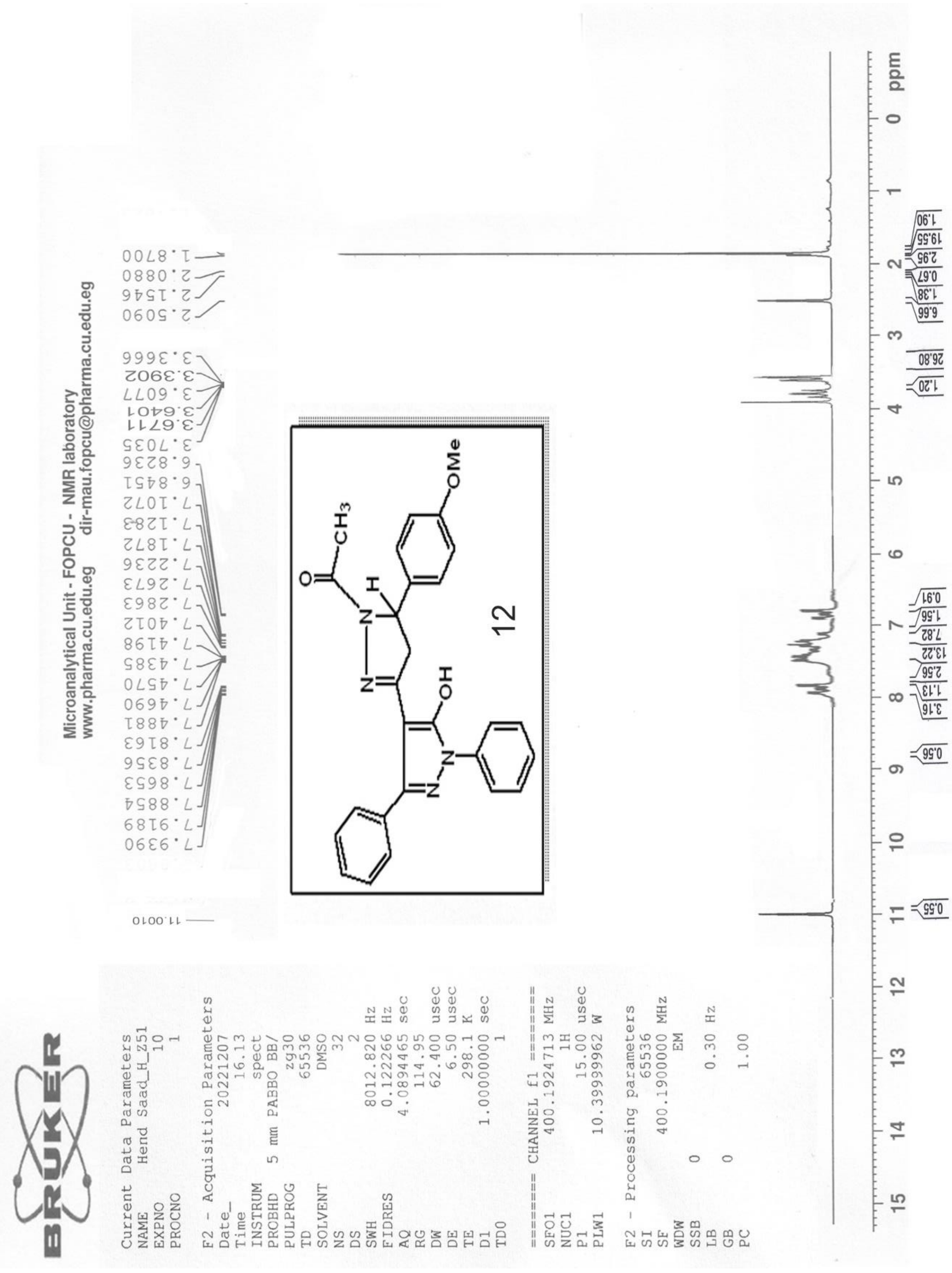

Figure S38: ^{1}H-NMR Spectrum of Compound (12) …. (DMSO)

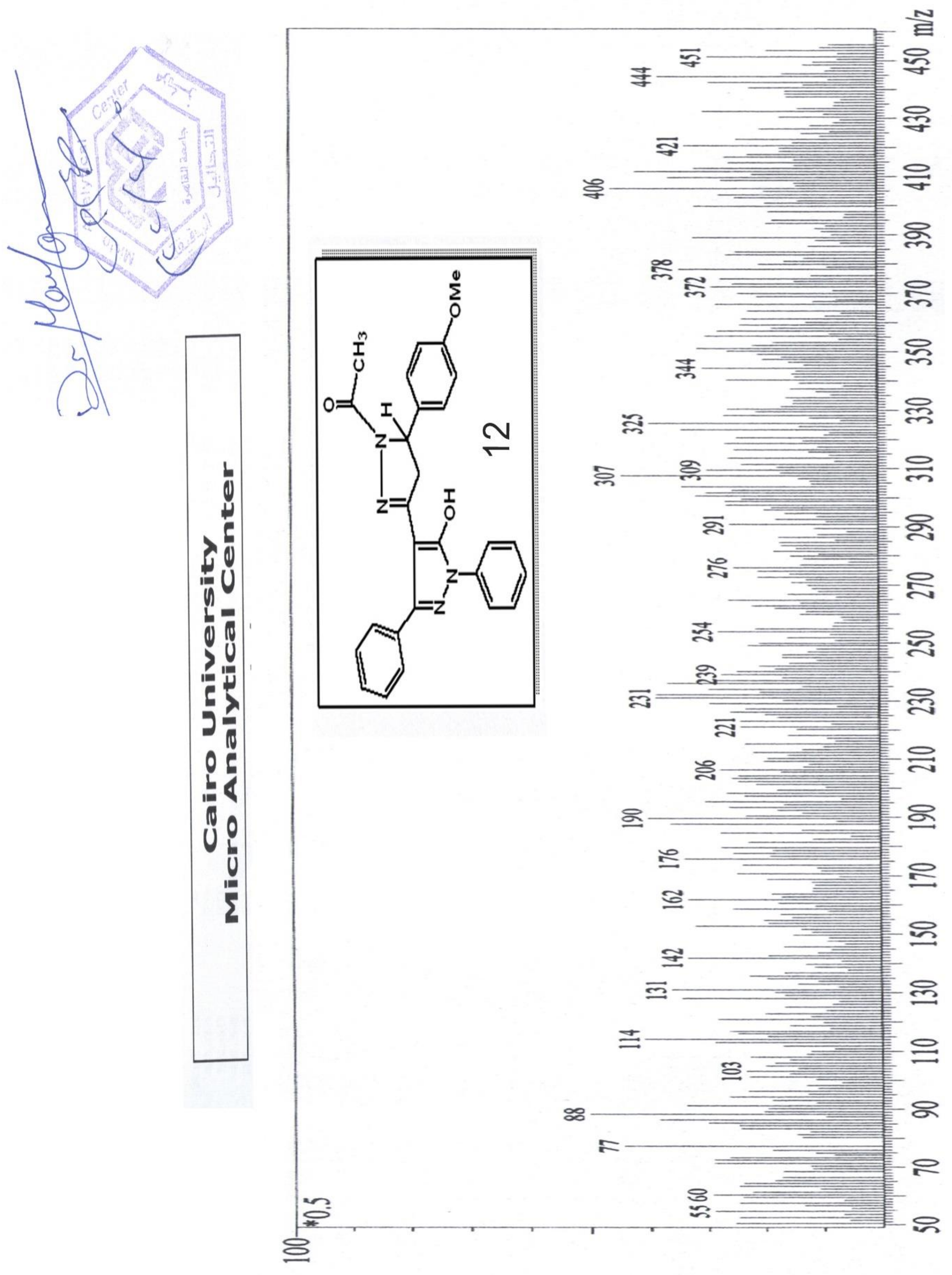

Figure S39: Mass Spectrum of Compound (12) M.wt=452 (M^+)

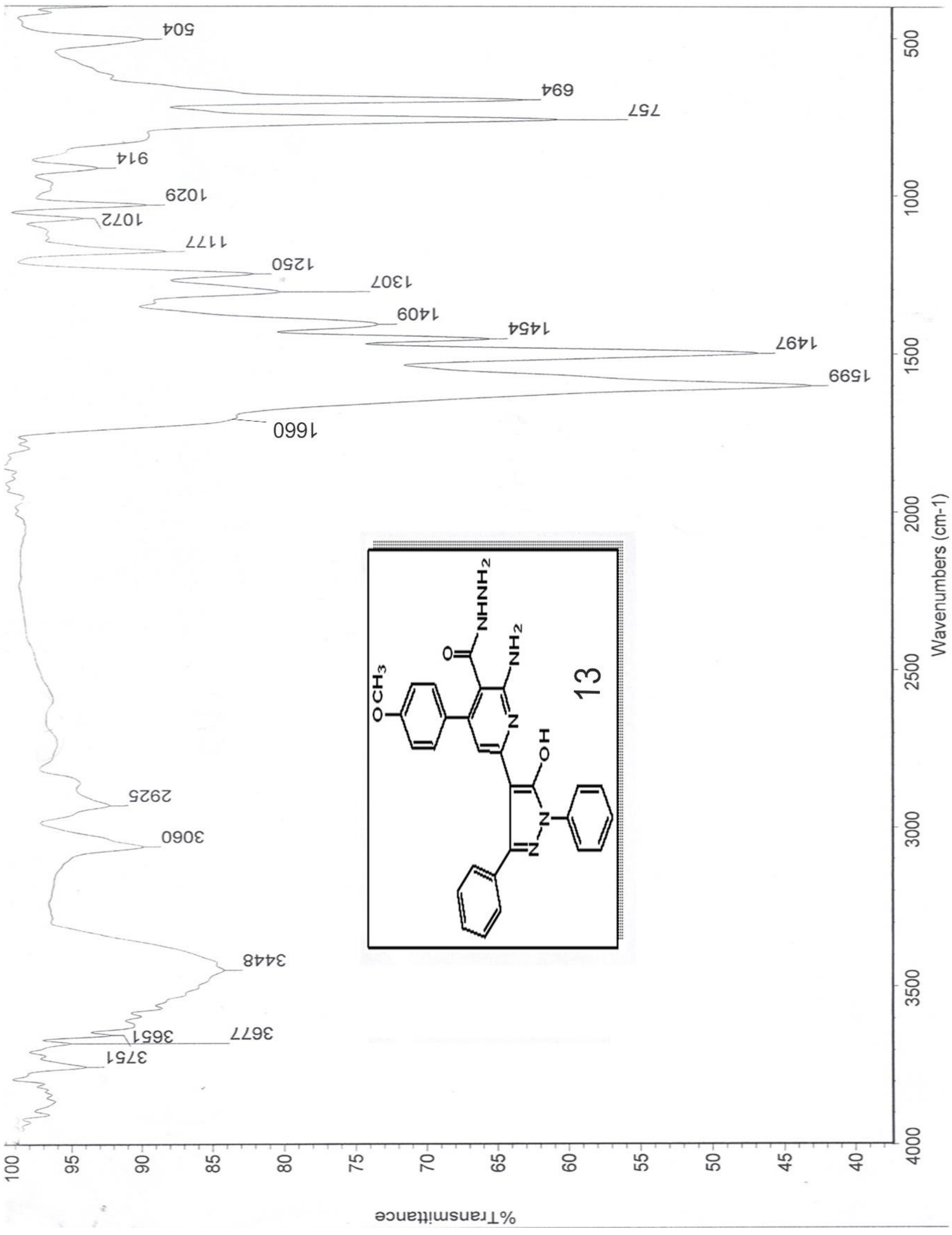

Figure S40: IR Spectrum of Compound (13)

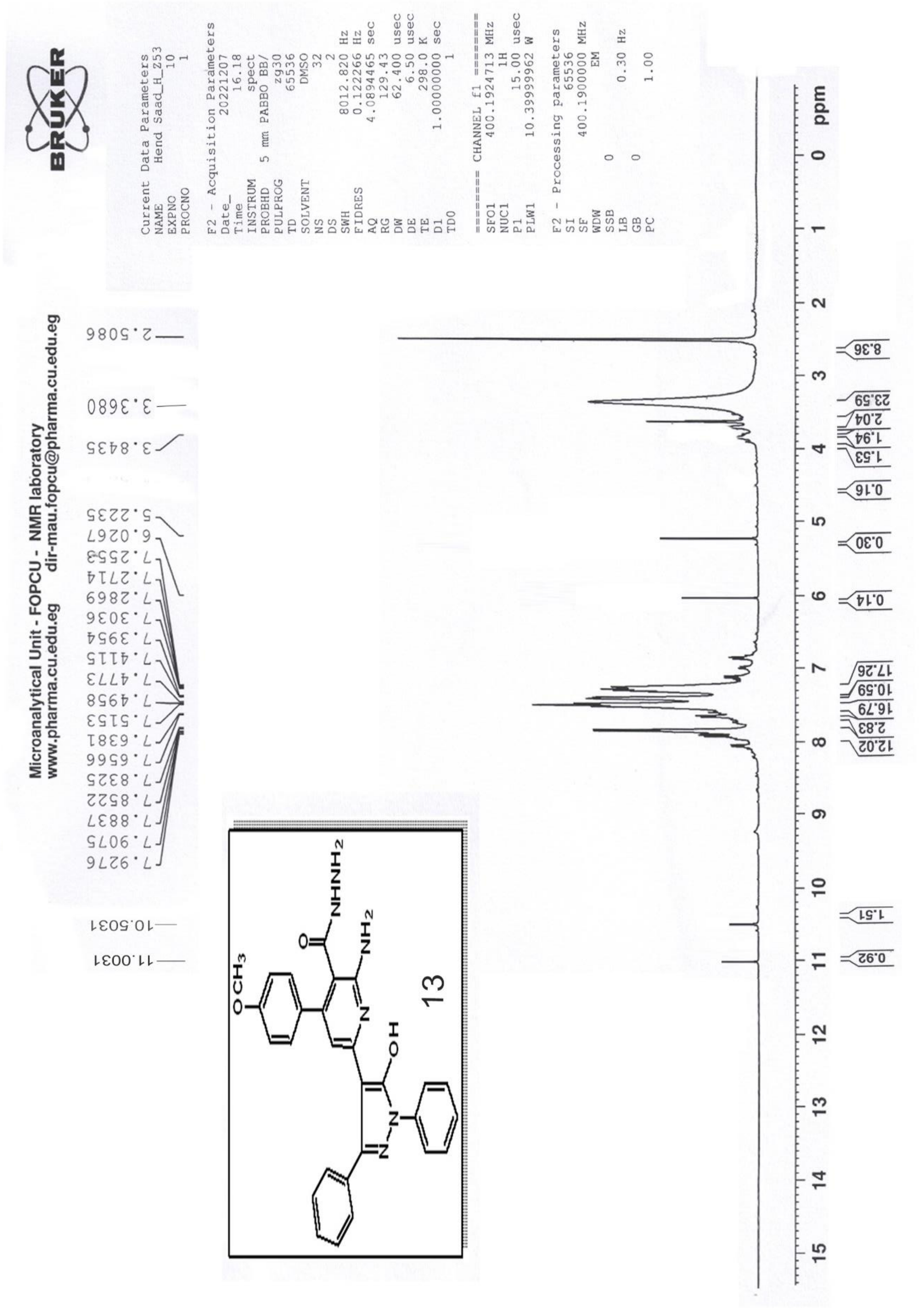

Figure S41: ^{1}H-NMR Spectrum of Compound (13) …. (DMSO)

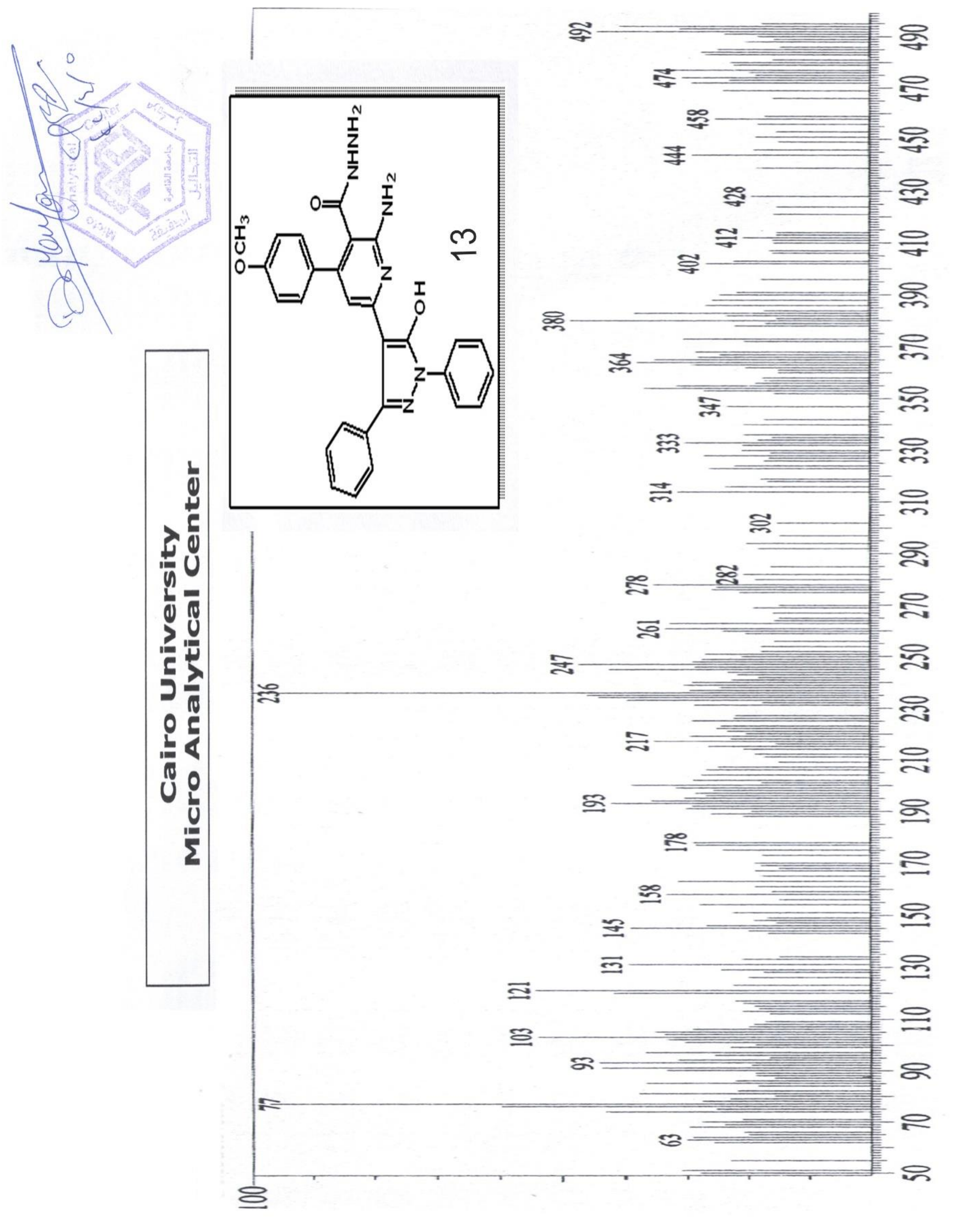

Figure S42: Mass Spectrum of Compound (13) M.wt=492 (M^+)

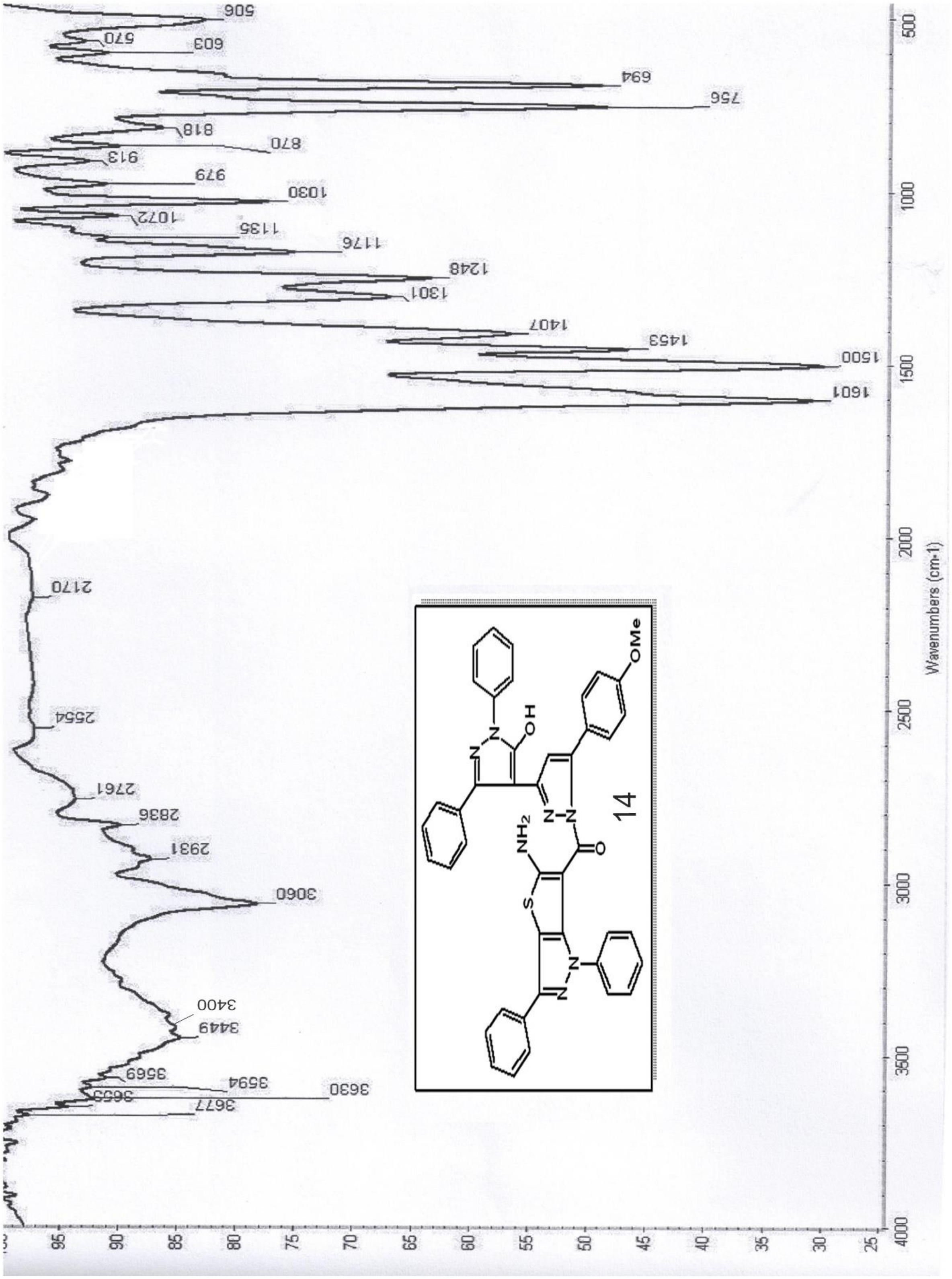

Figure S43: IR Spectrum of Compound (14)

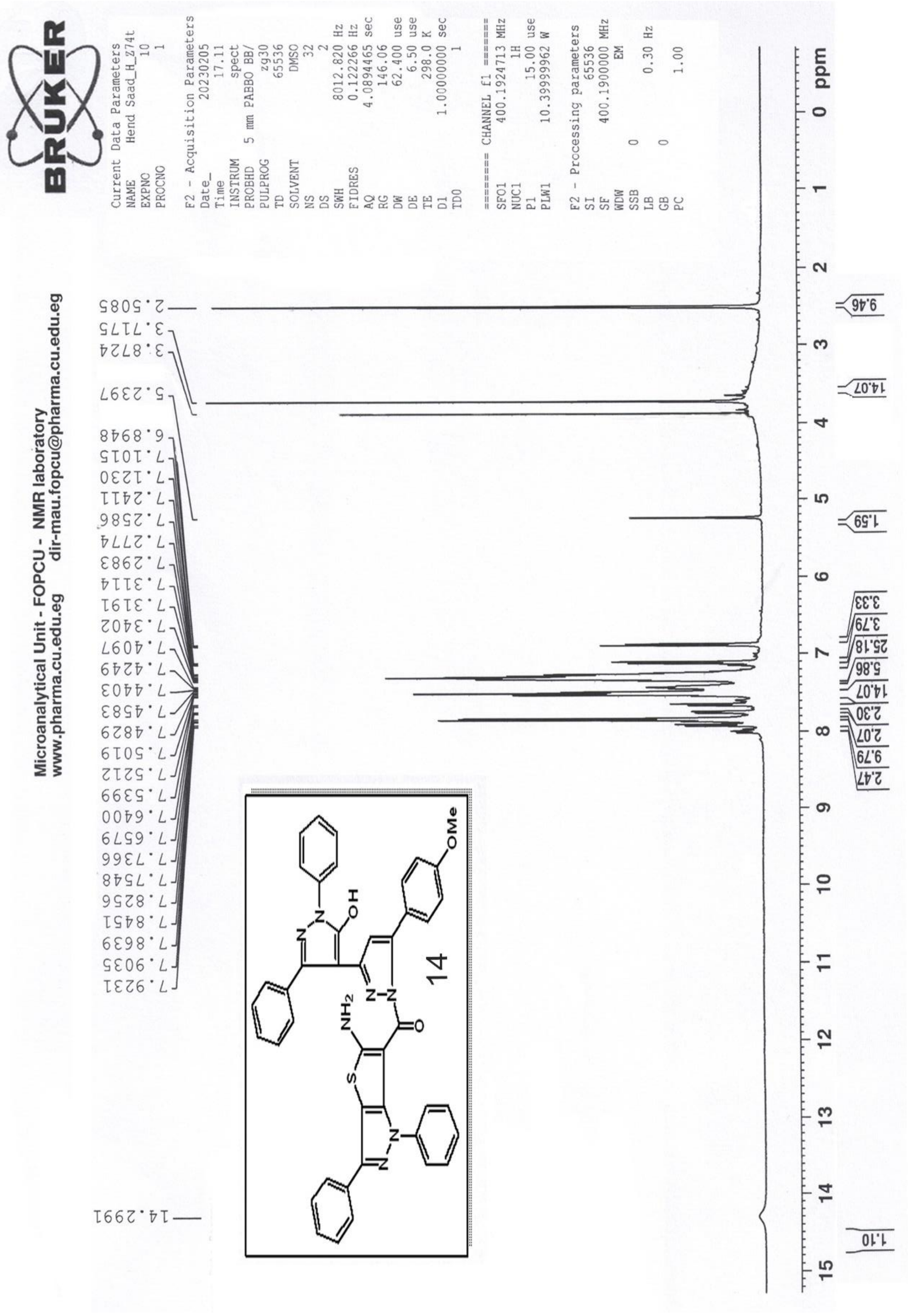

Figure S44: ^{1}H-NMR Spectrum of Compound (14) …. (DMSO)

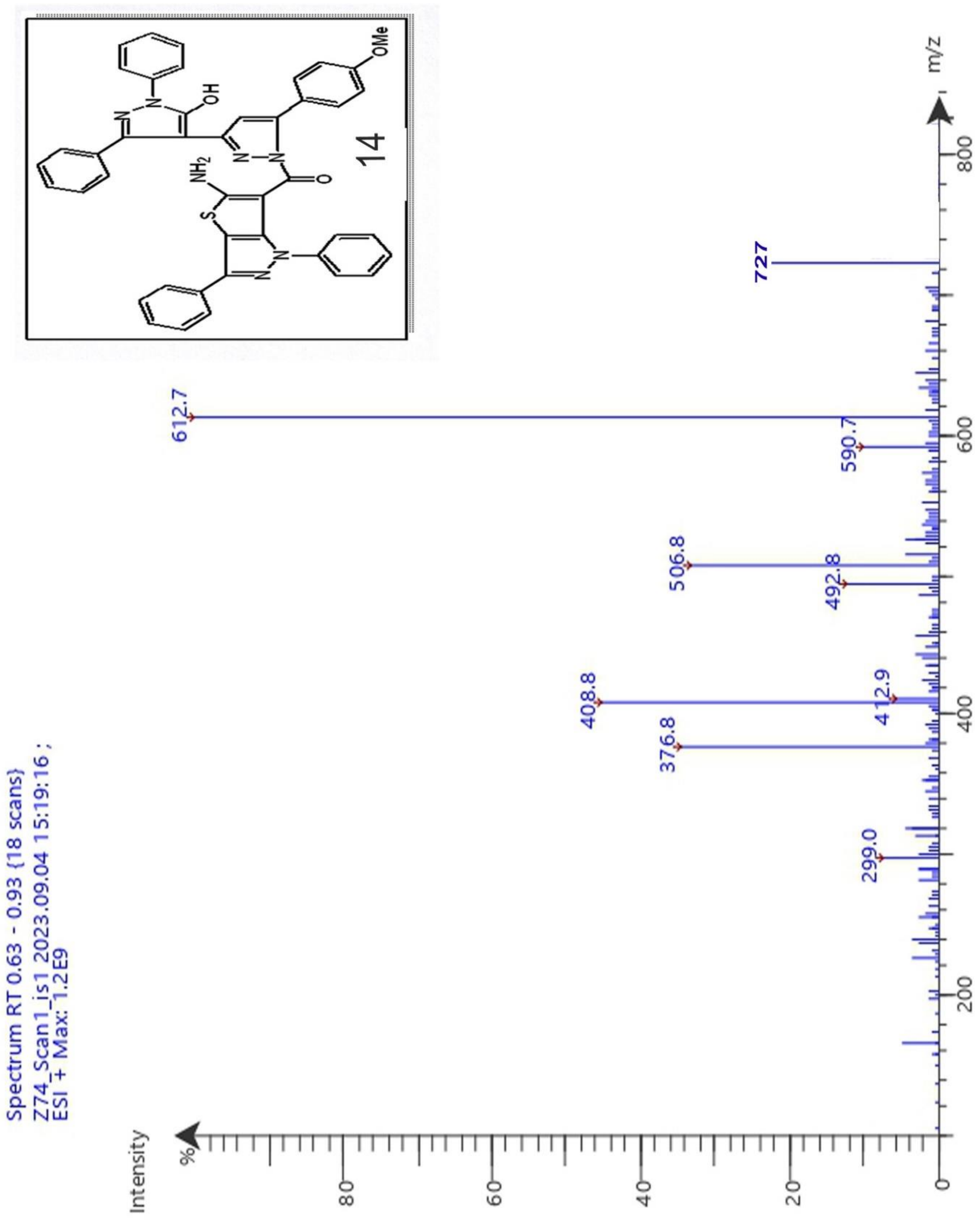

Figure S45: Mass Spectrum of Compound (14) M.wt=727 (M^{+}+2)

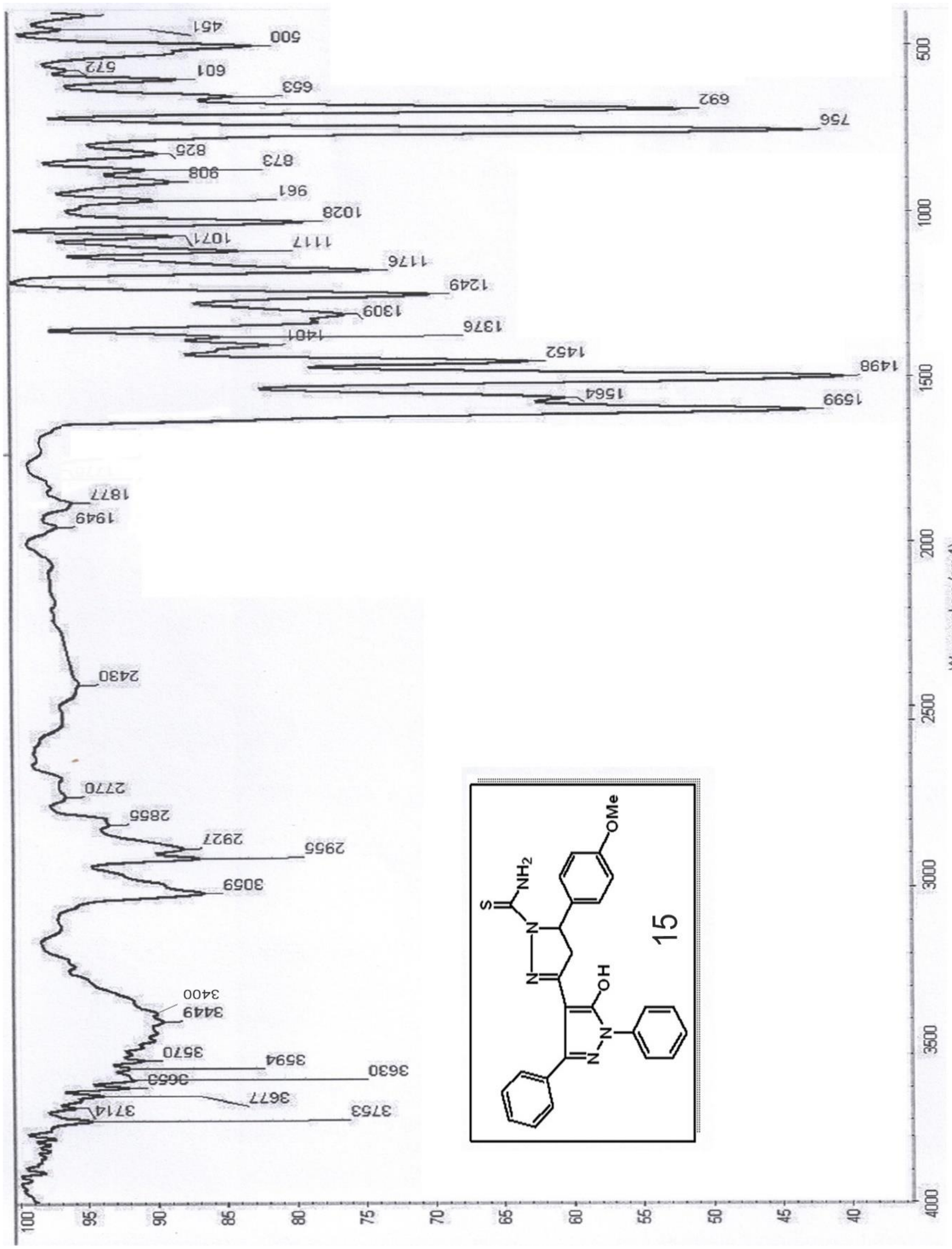

Figure S46: IR Spectrum of Compound (15)

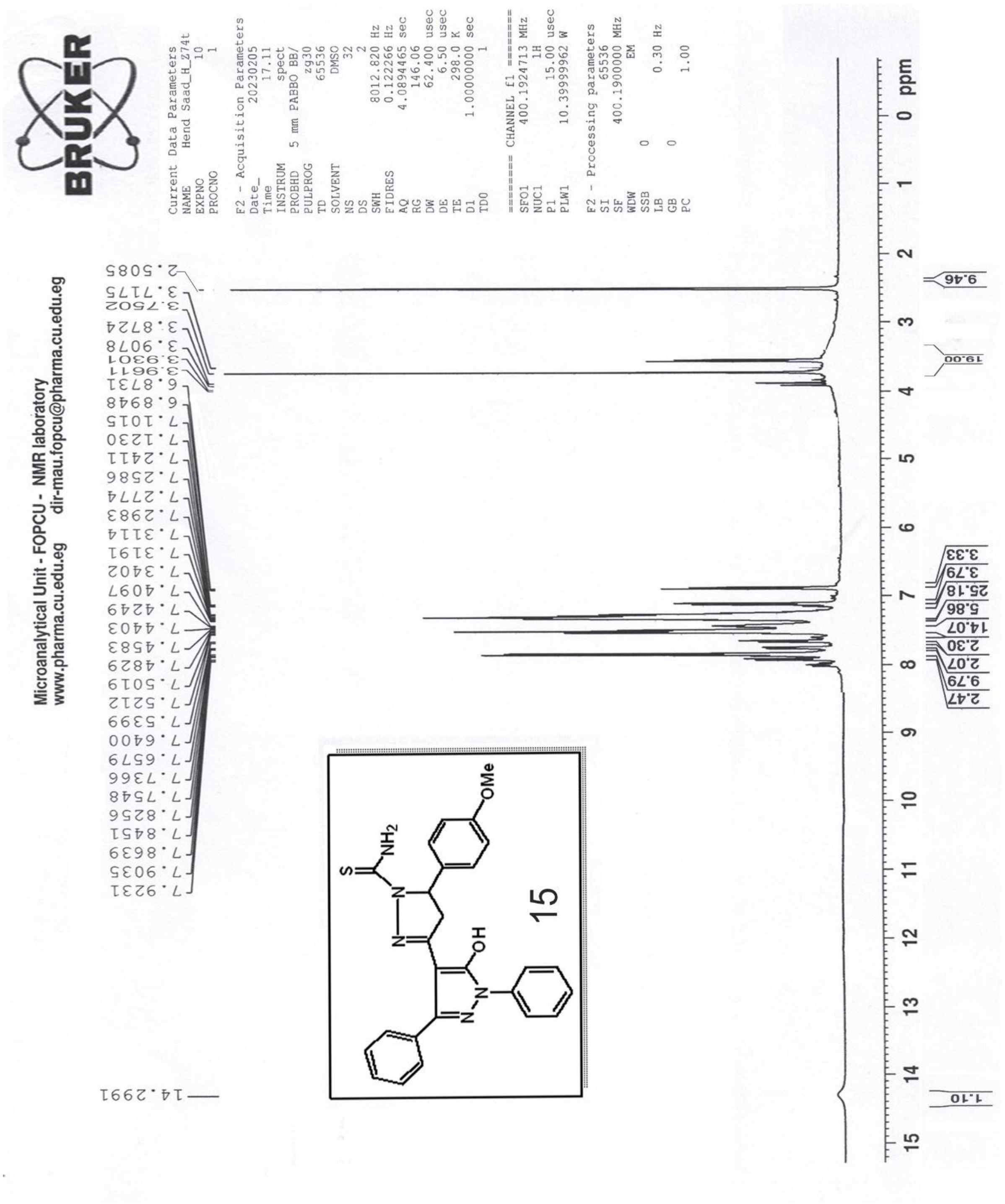

Figure S47: ^{1}H-NMR Spectrum of Compound (15) …. (DMSO)

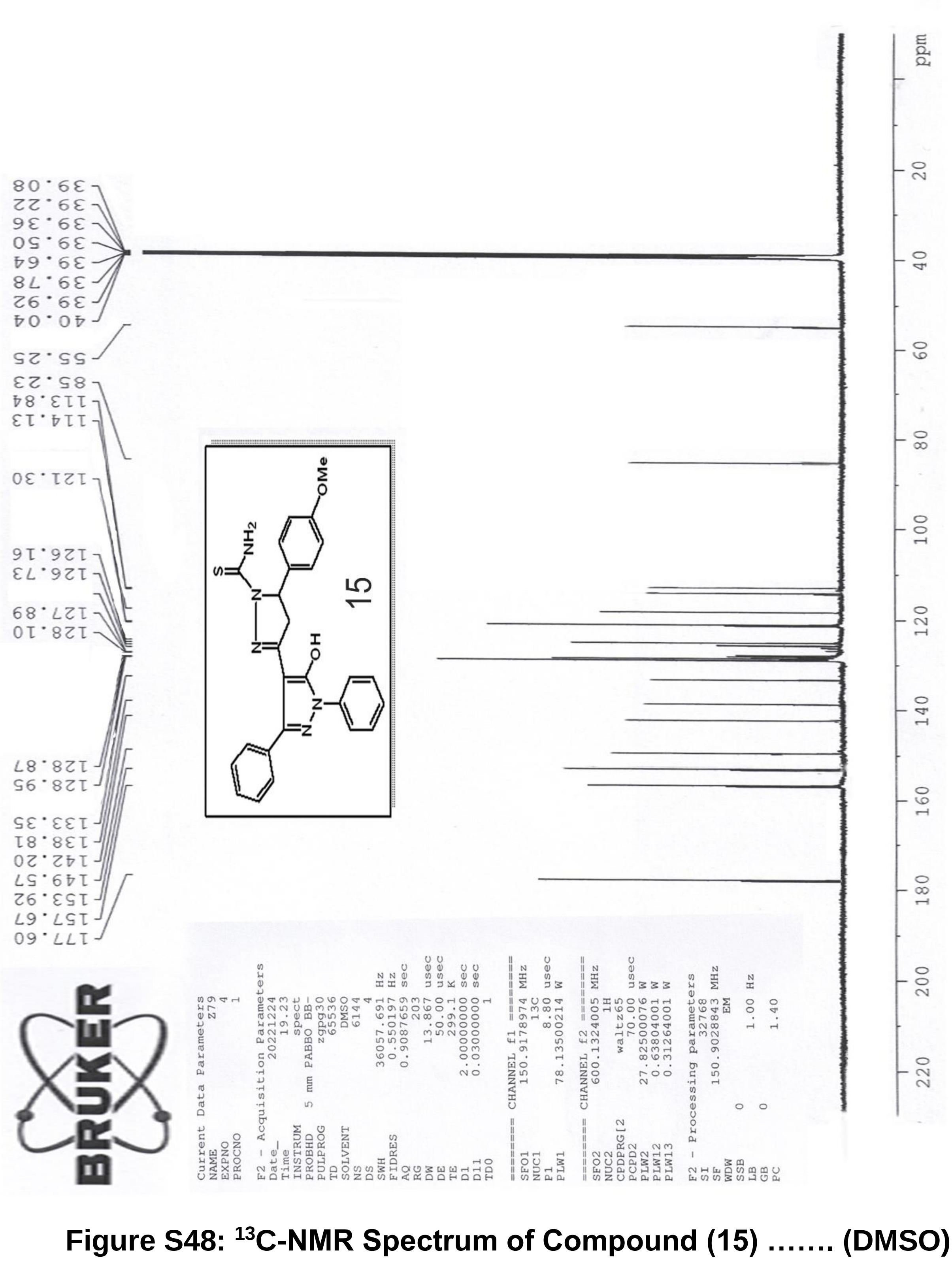

Figure S48: ^{13}C-NMR Spectrum of Compound (15) ……. (DMSO)
